# Transcriptomic analysis of a psammophyte food crop, sand rice (*Agriophyllum squarrosum*) and identification of candidate genes essential for sand dune adaptation

**DOI:** 10.1186/1471-2164-15-872

**Published:** 2014-10-07

**Authors:** Pengshan Zhao, Salvador Capella-Gutiérrez, Yong Shi, Xin Zhao, Guoxiong Chen, Toni Gabaldón, Xiao-Fei Ma

**Affiliations:** Key Laboratory of Stress Physiology and Ecology in Cold and Arid Regions, Gansu Province, Cold and Arid Regions Environmental and Engineering Research Institute, Chinese Academy of Sciences, Lanzhou, 730000 People’s Republic of China; Shapotou Desert Research and Experimental Station, Cold and Arid Regions Environmental and Engineering Research Institute, Chinese Academy of Sciences, Lanzhou, 730000 People’s Republic of China; Bioinformatics and Genomics Programme, Centre for Genomic Regulation (CRG), Dr. Aiguader, 88, 08003 Barcelona, Spain; Universitat Pompeu Fabra (UPF), 08003 Barcelona, Spain; Yeast and Basidiomycete Research Group, CBS Fungal Biodiversity Centre, Uppsalalaan 8, 3584 LT Utrecht, The Netherlands; Institució Catalana de Recerca i Estudis Avançats (ICREA), Pg. Lluís Companys 23, 08010 Barcelona, Spain

**Keywords:** *Agriophyllum squarrosum*, Sand rice, Salt tolerance, Heat tolerance, Comparative transcriptomics, Wild plant domestication

## Abstract

**Background:**

Sand rice (*Agriophyllum squarrosum*) is an annual desert plant adapted to mobile sand dunes in arid and semi-arid regions of Central Asia. The sand rice seeds have excellent nutrition value and have been historically consumed by local populations in the desert regions of northwest China. Sand rice is a potential food crop resilient to ongoing climate change; however, partly due to the scarcity of genetic information, this species has undergone only little agronomic modifications through classical breeding during recent years.

**Results:**

We generated a deep transcriptomic sequencing of sand rice, which uncovers 67,741 unigenes. Phylogenetic analysis based on 221 single-copy genes showed close relationship between sand rice and the recently domesticated crop sugar beet. Transcriptomic comparisons also showed a high level of global sequence conservation between these two species. Conservation of sand rice and sugar beet orthologs assigned to response to salt stress gene ontology term suggests that sand rice is also a potential salt tolerant plant. Furthermore, sand rice is far more tolerant to high temperature. A set of genes likely relevant for resistance to heat stress, was functionally annotated according to expression levels, sequence annotation, and comparisons corresponding transcriptome profiling results in *Arabidopsis*.

**Conclusions:**

The present work provides abundant genomic information for functional dissection of the important traits in sand rice. Future screening the genetic variation among different ecotypes and constructing a draft genome sequence will further facilitate agronomic trait improvement and final domestication of sand rice.

**Electronic supplementary material:**

The online version of this article (doi:10.1186/1471-2164-15-872) contains supplementary material, which is available to authorized users.

## Background

Food security is one of this century’s key global challenges. Food system is precarious in the face of increasing global human population, changing consumption patterns, ongoing climate change and soil degradation, and growing scarcity of water and land [1–5]. The pace of global warming is expected to accelerate; while extreme weather events will become more frequent and the temporal and spatial precipitation is likely to be unpredictable [2, 5]. To date, wheat, maize, rice, and soybean are the main sources for human and livestock calories in many agricultural regions around the world [1]. The time lags of these crops in adapting to a changing environment urge scientists to exploit crop wild relatives as a new genetic resource in crop improvement [3, 4]. Furthermore, one recent assessment of national food supplies worldwide demonstrated that the diversity of crop species is reducing and global food homogeneity is increasing over the past 50 years, which are potential threats to food security [6]. Thus, developing new crop species and increasing crop diversity will be a cornerstone of sustainable and intensified food production.

Desert ecosystem is far more harsh than most agricultural ecosystems, where major crop species are grown. In the desert, the plant species diversity is low and new evidence has shown that speciation in such environments is extensively driven by recent climate and habitat changes [7, 8]. Plants survived in the desert regions must cope with challenging environmental factors, such as extremes of temperature, high evaporation, low and erratic precipitation, salinity, solar radiation, and high light intensity [9, 10]. The desert plants therefore fascinate scientists with their unique adaptation and survival strategies [11]. Recently, next-generation sequencing (NGS; i.e. genomics and transcriptomics) was used to explore the possible adaptation mechanism of some desert plants, i.e. *euphratica*[12, 13], *Rhazya stricta*[10], *Reaumuria soongorica*[14], and *Ammopiptanthus mongolicus*[15, 16]. A feature of these plants is extensive adaptation, and indeed such studies improve our understanding of how they adapt to various kinds of stress factors. However, the intrinsic adaptation and survival strategies throughout the different stages of the life cycle of these desert species do not lend themselves easy to transfer to our major crop plants. Domestication of a potential crop species from desert environments, which is able to buffer the ongoing climate change, promises to be an effective strategy to keep food security.

**Figure 1:**
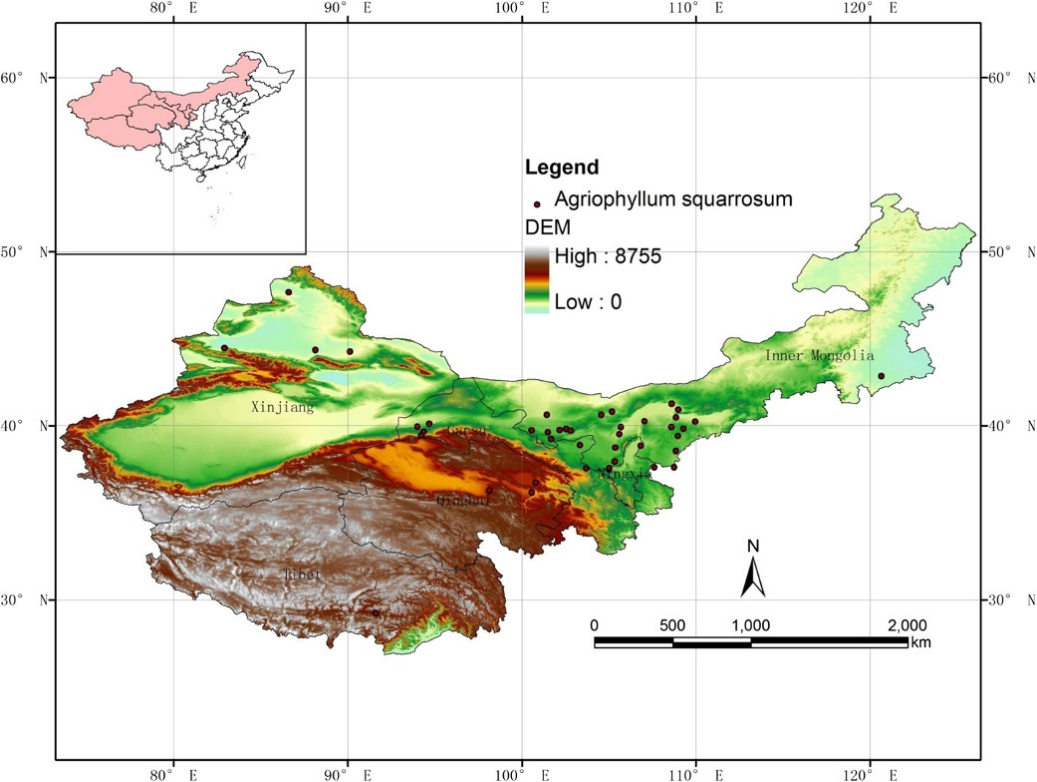
**Distribution of Sand rice in northern China.** The map was carried out based on altitude by ArcGIS software (http://www.esri.com/software/arcgis/). Red dots represented our sampling sites during wild survey. The latitude and longitude of each dot were listed in Additional file 1.

Sand rice (*Agriophyllum squarrosum*) is a pioneer annual psammophyte. This species and another important crop sugar beet (*Beta vulgaris*) are assigned to the Amaranthaceae family within the order of Caryophyllalles. Sand rice intrinsically adapts to the mobile and semi-mobile sand dunes in arid regions of China (Figure 1 and Additional file 1). The distribution areas also include Mongolia, Central Asia, and Russia. The nutrition values of sand rice seeds are comparable with the Amaranthaceae species quinoa [17], and archaeological records showed that sand rice has been used as army provisions in Tang Dynasty (AD 618–907) in China [17, 18]. Thus, sand rice represents a new crop alternative for future food production, yet its resilience and adaptive capacity remain largely unexplored and poorly understood. We here conduct transcriptomic analysis to dissect the genetic mechanisms that enable sand rice to adapt to desert environments. The sequence information will guide our subsequent domestication of this plant to cope with future food security.

## Results

### Annotation and functional characterization of sand rice unigenes

Total RNA was isolated from roots, mature leaves, inflorescences, and stems of single plant (Additional file 2). The sample was sequenced on a single lane of the Illumina HiSeq 2000 platform. After stringent quality assessment and data filtering, 30,283,868 reads (6.1 G) with 86.88% Q30 bases were yielded (Additional file 3). The raw paired-end sequence dataset was deposited in the National Center for Biotechnology Information (NCBI) Short Read Archive under accession number SRR1559276.

**Figure 2:**
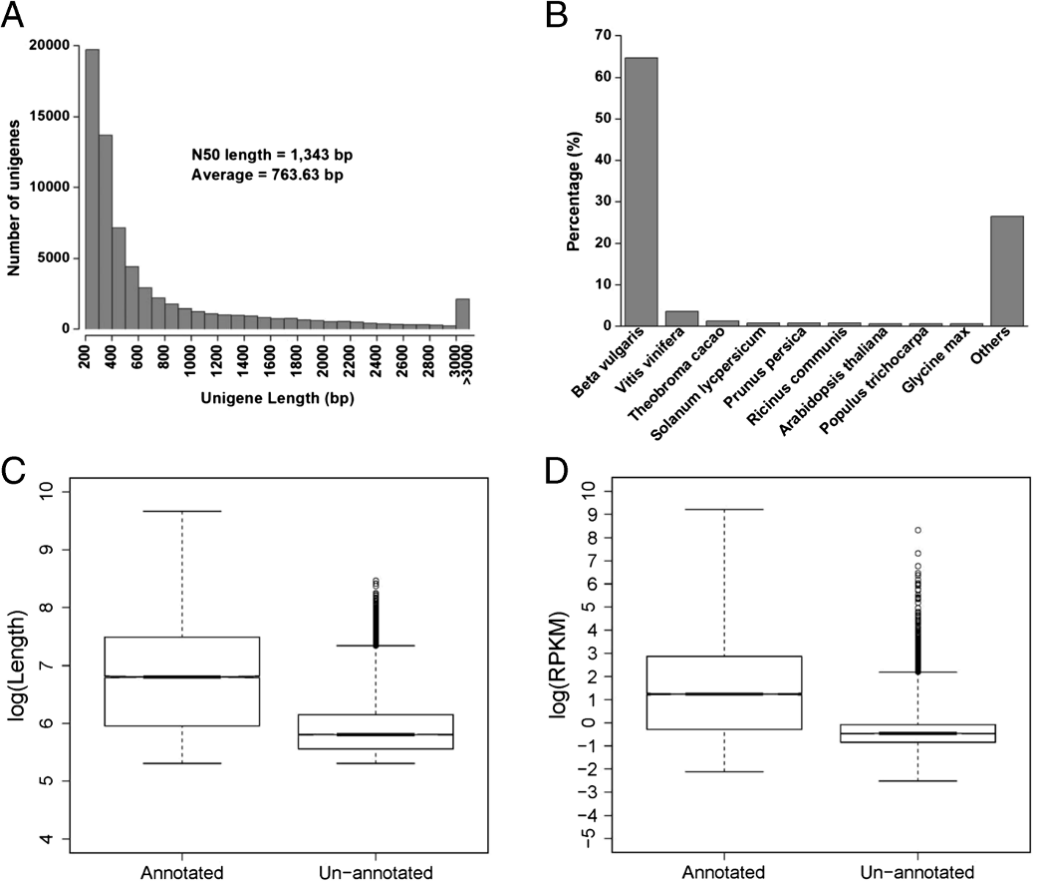
**Analysis of the sand rice trancriptome. (A)** Histogram of the length distribution of assembled unigenes. **(B)** Taxonomic distribution of the top BLAST hits for each unigene. **(C)** Length distribution of annotated and un-annotated unigenes was inferred with log(Length). **(D)** Expression level of annotated and un-annotated unigenes was inferred by log(RPKM).

The clean reads were assembled by Trinity program (Table 1; [19]) and 104,118 transcripts were generated with average length of 1107 bp and an N50 length of 1950 bp (Additional file 4). These transcripts were further subjected to cluster and assembly analyses. A total of 67,741 unigenes was obtained with mean length of 764 bp and an N50 value of 1343 bp (Figure 2A). The length of a given unigene was positively correlated with the number of reads assembled into it, which is expected for a randomly fragmented transcriptome (Additional file 4). Furthermore, Open Reading Frames (ORFs) were analyzed by getORF from EMBOSS package and 67,458 unigenes (99.58%) had ORFs with a start codon (Additional files 4 and 5). These results demonstrate a high coverage over this species transcriptome. To validate the assembly quality of sand rice unigenes, 18 unigenes were randomly selected to perform RT-PCR and 16 of them were successfully amplified (Additional file 6), suggesting that the assembled unigenes were highly accurate.

**Table 1:** **Overview of** ***de novo*** **sequence assembly for sand rice**

Length range (bp)	Contigs	Transcripts	Unigenes
200-300	1,351,306 (96.03%)	21,597 (20.74%)	19,711 (29.10%)
300-500	24,424 (1.74%)	23,915 (22.97%)	20,837 (30.76%)
500-1000	15,609 (1.11%)	19,725 (18.94%)	12,693 (18.74%)
1000-2000	10,162 (0.72%)	21,023 (20.19%)	8,679 (12.81%)
>2000	5,688 (0.40%)	17,858 (17.15%)	5,821 (8.59%)
Total number	1,407,189	104,118	67,741
Total length	126,635,518	115,271,030	51,729,464
N50 length	136	1,950	1,343
Mean length	89.99	1107.12	763.63

To further characterize the sand rice transcriptome, GC content of unigenes for sand rice and of transcripts for *Arabidopsis*, soybean, and rice were computed (Additional file 7). The GC content of approximately 59% of unigenes was in the range of 35%-45% (Additional file 7). The average GC content of sand rice was approximately 39.5% and slightly lower than that of *Arabidopsis* and soybean [20].

The entire unigenes were then aligned to the NCBI non-redundant protein (Nr) database, the Swiss-Prot protein database, and Clusters of Orthologous Groups of proteins (COG) with a threshold less than 1E-5. To increase the annotated unigenes number, a BLASTx comparison of the sand rice unigenes with the newly sequenced sugar beet peptide sequences [21] was conducted, and the results were incorporated into the Nr annotation results (See Methods). Among the 67,741 unigenes, 29,048 (42.88%) unigenes were significantly matched to the deposited ones in the public protein databases (Table 2 and Additional file 8). Approximately 65.50% of the unigenes were mapped to known genes in plants with best hits (E-value < 1e-50; Additional file 9), and 48.83% of unigenes can hit deposited sequences with similarity over 80% in Nr database (Additional file 9). The taxonomic distribution based on Nr annotations showed that 18,677 (64.58%) unigenes had top hits to *B. vulgaris* (Figure 2B). The technical limitation, such as read length and sequencing depth, affects the rate of transciptomic annotation to some extent [14, 22, 23]. The average length of un-annotated sequences was indeed shorter than that of the annotated unigenes (Figure 2C; 413 bp vs 1165 bp) and the expression level of un-annotated unigenes inferred by reads per kilobase per million reads (RPKM) was also much lower (Figure 2D). These results might be a simple explanation why only 43% unigenes were annotated. However, the possibility that some of these unigenes might be species specific genes cannot be rule out, because there were 734 unigenes (length ≥ 500 bp and RPKM ≥ 3) and 138 unigenes (length ≥ 1000 bp and RPKM ≥ 5) failed to hit any genes in public databases, respectively.

**Table 2:** **Summary of sequence annotation for sand rice**

Annotated databases	Unigene	Percentage (%)
Nr	28,920	42.69
Swissprot	20,730	30.60
GO	22,270	32.88
COG	9,728	14.36
KEGG	5,848	8.63
Total	29,048	42.88

Annotations and associated cellular component, molecular function, and biological process gene ontology (GO) terms were carried out for each sand rice unigene (*E*-value < 1E-5). The summary of final sand rice transciptomic annotation and associated GO terms from this analysis was provided in Additional file 8. In total, 32.88% of unigenes (22,270) had a significant hit in the public databases and 22,227 unigenes received at least one GO term. The most abundant biological process GO terms were oxidation-reduction process (1,608 unigenes) and response to salt stress (1,381 unigenes). GO terms associated with response to other environmental cues, such as water deprivation (904 unigenes), abscisic acid (ABA, 855 unigenes), cold (855 unigenes), wounding (797 unigenes) and heat (465 unigenes), were also enriched. The highly enriched classifications in biological process GO terms suggest that most of annotated unigenes are involved in fundamental responses to environmental circumstance. The top 50 represented GO terms were shown in Additional file 10.

### Phylogenetic position of *A. squarrosum*and comparative transcriptomics of sand rice versus sugar beet

**Figure 3:**
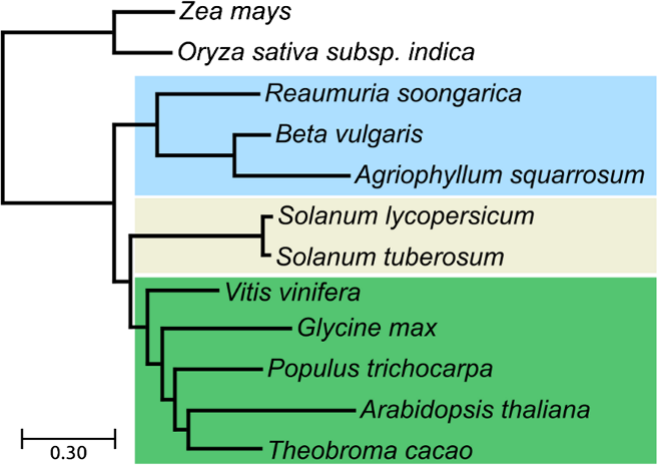
**Species phylogeny of ten fully-sequenced plant species plus the two new caryophyllales with transcriptomic data.** The phylogeny was based on the concatenation of 221 sets of one-to-one orthologous genes among the species used in this study. All aLRT branch support values were equal to 1.0 and, therefore, not shown in the tree. Different colour backgrounds highlights the relationships of the three main groups studied here: caryophyllales (light blue), asterids (Beige), and rosids (green). The placement of caryophyllales as basal to the branching of asterids and rosids was significantly more supported than an alternative placement as sister group of rosids.

After the publication of the first complete genome of a caryophyllales species, the sugar beet *B. vulgaris*[21], there is an increasing interest in understanding the phylogenetic position of other caryophyllales and the evolutionary relationships of this clade with other plant orders. The position of this order of flowering plants has been under debate as earlier work placed it with rosids, asterids or as sister branch to both groups [21, 24, 25]. Sugar beet remains the only fully-sequenced genome within this order, but the availability of large-scale transcriptomic data paves the way to increase the taxonomic sampling of phylogenomic analysis. Here we employed transcriptomic data coming from sand rice and another caryophyllales species *R. soongorica*[14] to elucidate their phylogenetic position. The use of transcriptomic data for phylogenomic analysis is challenging given the high fragmentation of sequenced genes, the presence of untranslated region, and the difficulty of establishing orthology relationships [26]. We initially searched for orthologs in *A. squarrosum* and *R. soongorica* of the 110 widespread marker genes that had been previously in a phylogenetic placement of *B. vulgaris*, concatenated these into a combined alignment, and performed Maximum Likelihood analyses (see Methods). However, this analysis rendered inconclusive results with no topology being significantly more supported than alternative placements, indicating either a lack of sufficient phylogenetic signal or the presence of noise in the data. We then increased the marker gene set by allowing one of the species used in the *B. vulgaris* analysis to be missing. This increased the dataset to 221 alignments of orthologous gene sets, which we concatenated. In addition we applied a strict alignment filtering to reduce sequence heterogeneity. The resulting topology inferred by Maximum Likelihood (Figure 3), places sand rice within a clade with sugar beet, whereas *R. soongorica* is branching earlier within caryophyllales. The placement of caryophyllales as a sister group to a clade formed by rosids and asterids is significantly more supported than other alternative placements of caryophyllales.

**Figure 4:**
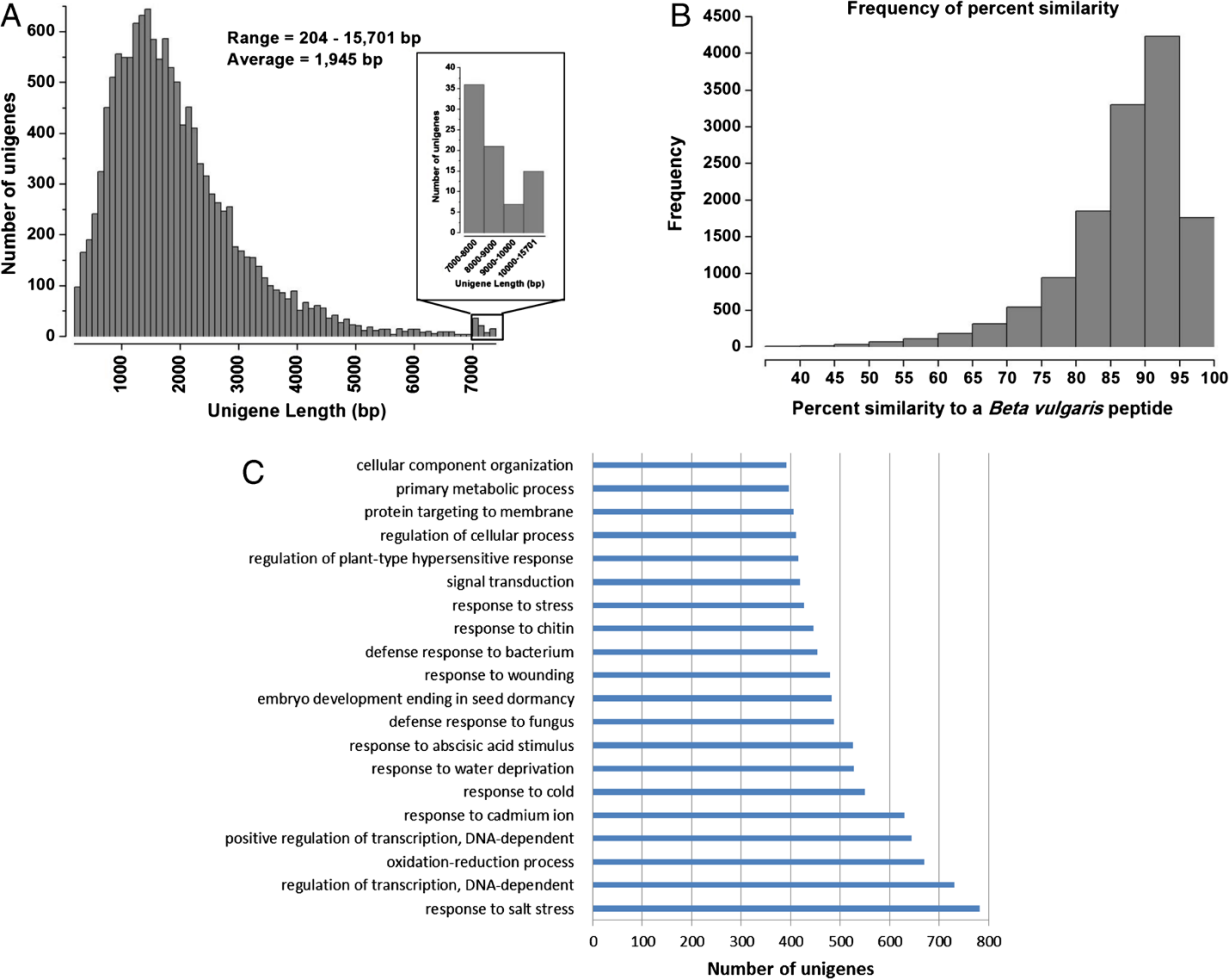
**Comparative analysis of sand rice unigenes versus** ***Beta vulgaris*** **genes. (A)** Histogram showing length distribution of sand rice unigenes with RBH. **(B)** Distribution of frequency versus percentage similarity (positive amino acid identity) of sand rice unigenes versus a *Beta vulgaris* peptide. **(C)** The 20 most highly represented BP GO terms of unigenes with RBH were shown.

To evaluate gene conservation between sand rice and sugar beet, we compared the assembled unigenes to sugar beet protein sequences (http://bvseq.molgen.mpg.de/Genome/Download/RefBeet-1.1/). A BLASTx comparison showed that 25,252 of the 67,741 sand rice unigenes had significant (*E*-value ≤ 1E-5) top hits to sugar beet peptide sequences (Additional file 11). A tBLASTn comparison was then performed between sugar beet and sand rice. We found that 23,876 peptide sequences had best hits to sand rice unigenes (Additional file 11). To reduce the chance of mistaking a paralogue for an orthologue, we identified as pairs of putative orthologs only those consisting of reciprocal best hits (RBH). This approach resulted in a total of 13,334 pairs of putative orthologs, each pair corresponding to a single sand rice unigene and a sugar beet peptide sequence. The length of approximately 71.31% of unigenes was in the range of 700 – 2500 bp and with an average length of 1945 bp (Figure 4A). The relative homology of each unigene to the most similar sugar beet peptide was measured by the percentage of positive sequence similarity (Figure 4B). A large proportion (90.60%) of unigenes showed more than 80% similarity to the corresponding sugar beet orthologue. In addition, a total of 11,581 unigenes were assigned at least one GO term. The most highly represented biological process GO terms were response to salt stress (782 unigenes) and regulation of transcription/DNA-dependent (731 unigenes). Sugar beet is a well–known salt resistance plant. Recent proteomic analysis showed that the differentially accumulated proteins after salinity treatment are mainly related to photosynthesis, protein folding and degradation, metabolism, and stress and defense [27, 28]. Out of the 782 unigenes, we identified 20 potential chaperons, seven aquaporins and six late embryogenesis abundant proteins (LEAs). The enzymes involved in scavenging reactive oxygen species (ROS), such as ascorbate peroxidase (APX), monodehydroascorbate reductase (MDAR), and glutathione S-transferase (GST), and in other metabolism processes were overrepresented. We also observed 110 and 38 unigenes encoded transcription factors and kinases, respectively. The 20 most highly represented biological process GO terms were shown in Figure 4C.

### Candidate genes involved in heat tolerance in sand rice

**Figure 5:**
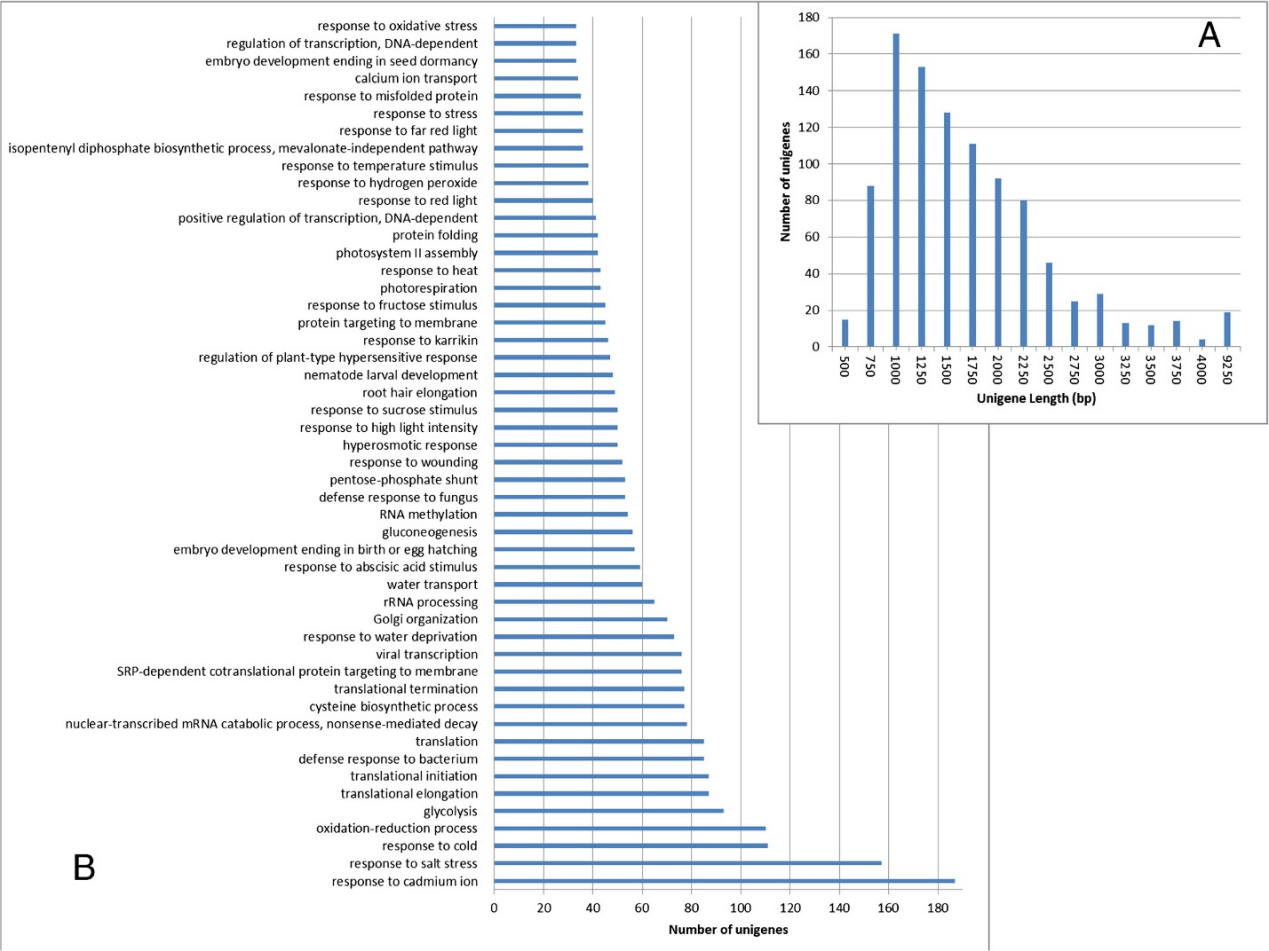
**Information of candidate genes in sand rice transcriptome. (A)** Histogram showing length distribution of the 1000 most highly expressed unigenes. **(B)** The 50 highly represented BP GO terms of unigenes were shown.

Detailed studies of various species have showed that variations in basal or constitutive expression strengths of stress-related genes enable an individual resilient to changing environments [29–33]. A specific term “frontloaded genes” was named for these constitutively expressed genes [29]. By comparing with their respective less-adapted species, the effect of so–called frontloaded genes on the niche adaptation has been highlighted in *Arabidopsis halleri*[34], *Alyssum lesbiacum*[35], and *Thlaspi caerulescens*[36]. Sand rice was far more resilient to high temperature than other plant species (Additional file 12). To dissect the stress tolerance mechanism and screen the possible responsible genes, the top 1,000 abundant unigenes inferred from the RPKM values were first analyzed. The length of 65.5% of unigenes was in the range of 1000–2000 bp (Figure 5A). A total of 938 unigenes was assigned at least one GO term and the 50 most highly enriched biological process GO terms were shown in Figure 5B. To further understand the function of these highly expressed unigenes, the corresponding *Arabidopsis* orthologs were searched with the same approach as the comparison of sand rice versus sugar beet and 725 RBH pairs were indentified (Additional file 13). We found that 26 unigenes encoded putative kinases and 19 of them had *Arabidopsis* orthologs. An *Arabidopsis* glycogen synthase kinase3 (ASKα), which is homologous to sand rice comp20065_c0, is critical for the regulation of redox stress response and tolerance of salt stress [37]. Also a gene coding for a sucrose nonfermenting 1-related protein kinase 2.4 (SnRK2.4; comp21292_c0 vs AT1G10940) was identified. SnRK2.4 plays an important role in mediating drought, salt, and osmotic stress signaling and tolerance [38–40] and is essential for root, shoot and pollen development [39, 41, 42]. New evidence demonstrated that SnRK2.4 is also involved in cadmium stress response by controlling ROS accumulation [43]. Among the 1000 most highly expressed unigenes, 24 unigenes encoded putative transcription factors and 21 of them had orthologs in *Arabidopsis* (Additional file 13). The main function of these transcription factors included mediation of abiotic stress responses and hormone responses and regulation of flowering and circadian rhythm. One of them (comp42160_c0 vs AT3G24500) encodes a transcriptional coactivator multiprotein bridging factor 1c (MBF1c), which is a key regulator of thermotolerance and controls ethylene–, glucose–, trehalose–, and salicylic acid–signaling pathways in *Arabidopsis*[44–46]. Mutant defective in MBF1c is sensitive to osmotic stress and oxidative stress [47]. MBF1c also controls leaf cell expansion through regulating the expression of endureduplication–related factors [48].

The daily and seasonal temperature in deserts fluctuates in a wide range. Anticipation of rising temperature early enough is crucial for plant cells to activate defense gene expression and accumulate so–called heat–shock proteins (HSPs) to survive against upcoming heat damage [49]. Interestingly, 43 of the 1000 most highly expressed unigenes were categorized into response to heat term and 12 unigenes encoded putative HSPs (Additional file 13). There are 4 HSP110s, 7 HSP100s, 7 HSP90s, 14 HSP70s, and 32 small HSPs in *Arabidopsis*[50] and 3 HSP110s, 4 HSP100s, 4 HSP90s, 4 HSP70s, and 18 small HSPs were identified with RBH in sand rice transcriptome (Additional file 13). Most of HSPs localized in cytosol based on the prediction by Finka et al. [50] and 12 HSPs were included into the top 1000 expression unigenes and seven were classified into response to heat GO term. Extensive studies in recent years have demonstrated that up-regulation of HSPs is regulated by a complex cascade and activation of heat-shock transcription factors (HSFs) are unquestionably the terminal steps to mediate the expression of HSP genes [49, 51]. *Arabidopsis* possesses diverse HSF families including 21 genes [51, 52]. Twelve of them had orthologs in sand rice transcriptome (Additional file 13). In *Arabidopsis*, four members belong to the HsfA1 subclass and HsfA1a, HsfA1b, and HsfA1d share the role of master regulator for triggering the expression of the heat stress response genes encoding chaperones and diverse transcription regulators, including HsfA2, HsfB1, Dehydration-responsive element binding protein 2A (DREB2A), MBF1c, and bZIP28 [51, 53–56]. Together with HsfA1, HsfA2 forms heterooligomeric complexes resulting in synergistic transcriptional activation of heat stress gene expression [51]. However, the orthologs of HsfA1a and HsfA1e were not identified in sand rice (Additional file 13).

Transcriptome profiling technologies, including microarray and high-throughput sequencing platforms, markedly accelerate our knowledge of molecular processes and networks involved in stress tolerance in *Arabidopsis*. Recently, a data mining method named machine learning was used to screen stress-related genes and 227 heat stress-related candidate genes and 87 heat stress-specific expressed genes were identified [57, 58]. To exploit the possibility that translate this knowledge to sand rice, the orthologs of these genes were searched in sand rice transcriptome data. We found a total of 169 unigenes with 141 and 46 unigenes homologous to heat stress–related and –specific genes, respectively (Additional file 13). There were 34 unigenes included in the 1000 most highly expressed category, suggesting that constitutively high expression of these unigenes are an effective strategy to cope with the reoccurring heat stress damage.

Desert plants usually have to encounter recurring or multiple environmental stresses including drought, extreme temperatures, salinity, solar radiation, and high light intensity. High evaporation rates in desert areas lead to water scarcity and in turn salinity components accumulate in the surface layers of the sand dunes. Accordingly, sand rice is routinely subjected to a combination of various abiotic stresses - drought, salinity, and high temperature - all at once in midday. Recent transcript profiling studies have shown that the molecular response of plants to multifactorial stress cannot be directly extrapolated from the response to single applied stress [59–63]. Sewelam et al. [62] identified 190 candidate genes which are essential for enhancing plant resistance to a combination of salt, osmotic and heat stresses. We found that 67 out of 190 genes had an orthologoue with RBH in sand rice transcriptome and six and three unigenes encoded HSPs and LEAs, respectively (Additional file 13). There were 16 unigenes included in the 1000 most highly expressed category, suggesting that these genes enable sand rice to minimize damage caused by stresses or to sustain the constantly harsh environmental challenges.

## Discussion

### The putative heat adaptation related genes in sand rice

Plants thriving in desert environment face various factors: unpredictable annual precipitation and its distribution and often presents with extensive quantities when occurs; temperature changes in a broad range, extremely high and low at daytime and nighttime, respectively; intensive radiation and other abiotic and biotic stresses [11, 64]. A combination of ecological and evolutionary strategies is usually selected by most of species to mitigate extinction risks from climate variability [65]. Increasing lines of evidence have showed that desert plants have evolved a unique complementary set of adaptation and survival strategies throughout the different stages of their life cycles [11]. Recent advances in high-throughput and comparative genomics are shedding light on the evolutionary mechanisms how plants adapt to extreme environment [66, 67]. Barshis et al. [29] found a list of genes showing constitutively higher expression in thermally resilient corals. Such situation were also found in metal/ions tolerant extremophyte plants [33–36], however, such genes were not screened by Barah et al. [68] when dissecting the diversity of heat stress transcriptional response among ten ecotypes of *Arabidopsis thaliana*. Sand rice is far more resilient to heat shock than other plants (Additional file 12). To uncover the possible mechanism underlying the thermal tolerance, we firstly focused on the 1000 most highly expressed unigenes. Through comparative transcriptomics, 26 putative kinases and 24 transcription factor were identified, and key regulators such as SnRK2.4 and MBF1c were also included (Additional file 13). GO annotation showed that unigenes involved in cadmium, salt, and cold stress responses were enriched. Although directly comparing expression (RPKM) across unigenes for quantification in the total RNA library is inappropriate, we indeed found 43 unigenes are response to heat stress (Figure. 5) and twelve out of 33 HSPs with homologs in *Arabidopsis* are categorized into the most highly expressed group. Furthermore, transcriptomic profiling technologies has been used to systemically dissect the genetic mechanisms of stress responses in *Arabidopsis* in recent years. By comparative transcriptomics, we identified 169 and 67 heat stress–related/–specific genes and multiple stresses–related genes in sand rice transcriptome and 34 and 16 unigenes were constitutively high expressed genes, respectively (Additional file 13). All these results suggest that the constitutively high expressed unigenes in sand rice transcriptome are candidate genes relevant for desirable adapted traits. To verify these predicted heat-related genes, we selected 8 candidates for quantitative RT-PCR (Additional file 14). The expression of *SnRK2.4*, *HsfA1b*, *HSPs* (comp41797_c1, comp42214_c0, and comp19571_c0), heat stress–related gene (comp19559_c0), and heat stress–specific genes (comp41797_c1, comp19571_c0, and comp36531_c0) was significantly induced after 3 h heat stress treatment and the fold changes ranged from 2.5 to 2500. The expression levels of multiple stresses-related gene comp19684_c0/lipid transfer protein 4 (LTP4), were similar between control and heat-treated leaves, which is consistent with previous report in *Arabidopsis*[62], suggesting that sand rice LTP4 is also a multiple stresses-specific gene. Subsequent studies on these candidate genes will facilitate to unravel the mechanism of adaptation to extreme temperature in sand rice.

We found that twelve of 21 *Arabidopsis* HSFs had homologs in sand rice transcriptome (Additional file 13). In *Arabidopsis*, HsfA1s are key heat stress regulators and are comprised by four members, which constitute two pairs of duplicated genes (*HsfA1a* vs *HsfA1d*; *HsfA1b* vs *HsfA1e*) diverged after a recent whole genome duplication [53, 54]. Coincidently, *HsfA1a* and *HsfA1e* were absent in sand rice (Additional file 13). A simply explanation is that HsfA1s class is in ancestral condition and no expansion of this family has happened, although the technical limits cannot be ruled out. Interestingly, there was only one unigene (comp30600_c0) showed RBH with the sugar beet TNL class resistance gene (Bv_22240_ksro, Additional file 11), indicating that our phylogenetic result is convinced and the presence of a single TNL class gene is a feature of Amaranthaceae in contrast to the expansion of this family in rosids and asterids [21]. Further experiments are needed to determine the ancestral evolutionary events.

### Sand rice is also a possibly salt–resistant plant

Sand rice and sugar beet both belong to the order of Caryophyllalles. Phylogenetic analysis (Figure 3) confirmed again the previous result that Caryophyllalles branched out before the separation of asterids and rosids [21]. Sand rice is close to sugar beet, whereas extreme xerophyte plant, *R. soongorica*, is branched off earlier within this same family. Sugar beet has an estimated genome size of 714–758 Mb, including 27,421 protein-coding genes [21]. Sand rice is also diploid with 2*n* = 18 chromosomes [69]. The genome of the sand rice is approximately 705 Mb (unpublished data). BLAST comparisons of sand rice and sugar beet showed that our sand rice transcriptome has high coverage of sugar beet protein sequences (Figure 4). All these results showed the close relationship between sand rice and sugar beet and the genomic information of sugar beet will be very helpful for our domestication of the sand rice.

Through comparative transcriptomics, we identified 13,334 pairs of putative orthologues between sand rice and sugar beet, in which 782 unigenes were categorized into response to salt stress GO term (Additional file 11). The genes functioned as chaperons, LEAs, protective enzymes, sugar transporters, and ion channels and the genes related to transcription and protein synthesis were highly represented. The classifications of these unigenes were similar to the sugar beet salt responsive protein groups, implying that sand rice and sugar beet share similar salt tolerance genes. To be mentioned, the MYB-, ethylene-responsive transcription factor (ERF)/DREB- and homeobox-leucine zipper protein-family transcription factors and serine/threonine-protein kinases and CBL-interacting protein kinases were enriched among 782 unigenes. Abscisic acid–, gibberellin–, auxin–, ethylene–signal related genes were also included. All these genes have been demonstrated as essential components in response to salt stress and other environmental stresses in *Arabidopsis*[70, 71]. Moreover, we observed a group of glycine-rich RNA-binding proteins (GRPs) in this category. *In Arabidopsis*, three GRPs (atRZ-1a, GR-RBP4, and GRP7) play a negative role during seed germination and seedling growth and GRP2 only affects seed germination under salt stress condition [72–75]. It seems that similar strategy regulating gene expression at the post-transcriptional level is utilized in sand rice and sugar beet to cope with environmental stresses.

*P. euphratica* is well-known salt tolerant desert species. The genetic mechanism underpinning its salt adaptation was deeply studied recently [12, 13]. The differentially expressed genes in salt-treated *P. euphratica* callus are mainly categorized into transport, transcription, cellular communication, and metabolism. Comparison of salt responsive genes between *P. euphratica* and another poplar (*P. tomentosa*) showed that more genes were classified into cation transporter, oxidoreductase activity, and response to abiotic stimulus [12], suggesting that specific genes are exploited to confer salt adaptation in desert poplar. Although it is not very suitable for directly comparing our unigene dataset with the salt responsive genes of *P. euphratica*, the terms such as metabolic process, oxidation-reduction process, regulation of transcription, and response to stimuli were indeed high-ly represented in our transcriptomic dataset (Additional file 10). Moreover, large number of unigenes was categorized into carbohydrate, amino acid metabolic pathways (Additional file 8), which is also similar with the results of *P. euphratica* to some extent [13]. However, comparative transcriptome analysis is required in further studies to dissect the salt adaptation mechanism in sand rice.

## Conclusions and perspectives

Sand rice is a potential crop alternative for future food production adapted to harsh climates. However, this species has undergone only little agronomic modifications through classical breeding partly due to the absence of genomic information. In this study, 67,741 unigenes were obtained by deep Illumina RNA sequencing and approximately 43% of unigenes were annotated. Phylogenetic analysis clearly resolved the relationship between sand rice and other sequence crops, and provided additional support for the divergence of Caryophyllalles prior to the split of asterids and rosids. Comparative transcriptomics showed a high level of conservation in terms of gene content and sequence similarity between sand rice and sugar beet. We also identified a set of heat stress responsive genes by comparing with expression profiles of the *Arabidopsis* orthologs. Our transcriptome will accelerate the dissection of genetic variations and illustration of gene expression and regulatory mechanisms of sand rice adapting to harsh desert environment, and also help us to unravel the controlling mechanism for weedy traits, i.e. long hypocotyl, abnormal growth of lateral branches, flowering at the same time, seed dispersal [17]. In addition, our ongoing sequencing of the sand rice genome and screening of chemical-induced mutants will pave the path to the domestication of sand rice in a far shorter time frame.

## Methods

### Ethics statement

*A. squarrosum* is widely distributed in arid regions of China and is not an endangered or protected species. No specific permits were required when we collected the samples for this study.

### Distribution of sand rice

The distribution areas of sand rice in northern China were investigated during the past two years. The sampling sites were shown as red dots in Figure 1, which was carried out by ArcGIS software based on the altitude information of five provinces in northern China (http://www.esri.com/software/arcgis/).

### Plant materials and RNA extraction

Leaves, stem, roots, and inflorescences of sand rice (Additional file 2) were sampled in wild field, Yiwanquan, Jingtai County, Gansu province, northwest of China (37°21^′^50″N, 104°08^′^37″E), where the average annual precipitation is 180 mm from 1950 to 2000 (http://www.worldclim.org/). All samples were immediately frozen in liquid nitrogen and stored at −80°C for later RNA extraction.

Total RNA from each tissue was extracted with Plant total RNA Kit (TIANGEN, Beijing, China). The concentration and quality of each RNA sample were determined by 1% agrose gel electrophoresis, NanoDrop 2000™ micro**-**volume spectrophotometer (Thermo Scientific, Waltham, MA, USA) and Agilent 2100 Bioanalyzer (Agilent Technologies, Santa Clara, CA, USA). Equal amounts of purified RNA from each of the four tissues were pooled together to construct the cDNA library.

### Library preparation and RNA-seq

The cDNA library was prepared according to the description by Shi et al. [14]. Briefly, poly**-**A mRNA was purified by magnetic Oligo (dT) beads and fragmented followed by cDNA synthesis. The cDNA fragments were blunt**-**ended and ligated to sequencing adaptors. The ligation products were then size-selected for an insert size of 200 bp, and enriched by PCR with specific adaptor primers. Finally, the library was subjected to sequence by the Illumina HiSeq™ 2000 platform using 101 bp paired**-**end reads. A total of 30.28 million reads was generated with 86.88% above Q30 (Additional file 3).

### *de novo*assembly and functional annotation

The clean reads were obtained after filtering adaptor sequences and reads with ambiguous ‘N’ bases and with a base quality less than Q30. Trinity was then used to assembly the clean reads into contigs (Table 1 and Additional file 4; [19]). According to the paired**-**end information and sequence similarity, the contigs were clustered and further assemblied into transcripts. Finally, the longest transcripts in each cluster and the singletons were combined together as total unigenes. RPKM for each unigene was computed to determine the unigene expression profiles [76]. The ORFs were predicted by the “Getorf” program from the EMBOSS software package (Additional file 4; http://emboss.sourceforge.net/apps/cvs/emboss/apps/getorf.html). Functional annotation was conducted by aligning the unigenes to public protein databases (NCBI Nr, Swissprot, COG, KEGG) using BLASTx with an E**-**value less than 1e-5. For the statistical summary of Nr annotation and taxonomic distribution in Figure 2, Table 2, and Additional file 9, an additional BLASTx result (sand rice vs sugar beet, see below) was included based on the scores and e-value. Gene ontologies were assigned to each unigenes using Blast2GO [77].

### Phylogenetic analysis

#### Detection of marker genes in transcriptomic data from A. squarrosum and R. soongorica

The 110 phylogenetic marker genes recently used to reconstruct the species phylogeny of the closely relative *B. vulgaris*[21], or an extended dataset of 221 marker genes (see next section) were searched in the transcriptomic data coming from two caryophyllales species *A. squarrosum* (this study) and *R. soongorica*[14], with 67,741 and 46,203 predicted unigenes respectively. This was done by scanning both transcriptomes using exonerate [78] with a progressive relaxation of the minimum percentage of the aligned region from 80% to 20% in steps of 5%. Varying such score allows to detect the orthologous sequences in both species. Additionally it readily identifies the coding part of the unigenes, which usually contain untranslated regions. Using this approach 99 and 100 orthologous sequences out of the 110 original marker genes, 208 and 182 orthologs out of the 221 for the extended markers set, were detected for *A. squarrosum* and *R. soongorica*, respectively, at different stringency levels. Of note for *R. soongorica*, 19 marker genes, 27 for the extended dataset, mapped to clusters of unigenes for which the longest sequence was selected as the representative of the cluster. The sequence data of the phylogenetic tree was deposited in TreeBase: http://purl.org/phylo/treebase/phylows/study/TB2:S16290.

#### Multiple sequence alignment of individual markers

Unigenes detected as orthologs were added to the individual multiple sequence alignments (MSA) used to reconstruct the species phylogeny of sugar beet [21]. Sequences were added to the original untrimmed MSA using mafft v7.13 [79] and then sites with residues only in the newly aligned sequences were removed. Finally trimAl v1.4 [80] was used to remove columns that were filtered in the original study [21].

#### Phylogenetic placement of A. squarrosum and R. soongorica

Filtered MSAs corresponding to 109 or 221 sets of marker genes including predicted orthologs in *A. squarrosum* and *R. soongorica*, were concatenated. For the 221-genes dataset an additional filtering step based BMGE program [81] was applied to reduce the data heterogeneity in the alignment as this may impact negatively on the inferred species tree [82]. BMGE, parameters were: −w 1 -h 1 -g 1 -s FAST. Species relationships were inferred from these (filtered) concatenated alignments using a Maximum Likelihood (ML) approach as implemented in PhyML v3.0 [83], using JTT as evolutionary model, which was the best fitting model in the majority of individual alignments. The tree topology search method was set to SPR (Subtree pruning and regrafting). Branch supports were computed using an aLRT (approximate likelihood ratio test) parametric test based on a chi-square distribution.

To compare statistical supports of the Maximum Likelihood topologies with alternative placement of caryophyllales as i) basal to both rosids and asterids or iii) sister group of rosids, we generated alternative topologies using ETE v2 [84] and their likelihoods were computed for the same alignment with the same parameters. Log-likelihoods of alternative topologies were compared using CONSEL [85] using the eight different statistical tests implemented in this program.

### Comparative transcriptome analysis

Identification of the orthologous sequences was performed firstly by BLASTx using assembled unigenes and sugar beet protein sequences (http://bvseq.molgen.mpg.de/Genome/Download/RefBeet-1.1/). The threshold is *E*-value ≤ 1E-5. To avoid the possibilities of mistaking a paralogue for an orthologue, a BLASTx comparison was then conducted to find the sand rice unigene with best hit to each sugar beet peptide sequence. Finally, the pairs of putative orthologs (i.e. sand rice unigene x and sugar beet protein X were consistently found as reciprocal best hit for each other) were identified. The same approach was also used to screen the orthologous sequences between sand rice and *Arabidopsis*, using sequences downloaded from the TAIR 10 release (http://www.arabidopsis.org). The detailed information was showed in Additional files 11 and 13.

### Heat shock treatment

The sand rice seeds were collected at Shapotou Desert Research & Experiment Station (SPT, 37°27^′^38″N, 104°59^′^59″E) and Naiman Desertification Research Station (NM, 42°59^′^23″N, 120°46^′^01″E), Cold and Arid Regions Environmental and Engineering Research Institute, Chinese Academy of Sciences. The average annual precipitation of SPT and NM is 186 mm and 350–500 mm, respectively. Sand rice seedlings at five-leaf stage were exposed to 50°C for 3 h in dark and then allowed to recover for 7 d. No significant phenotype was observed in the seedling of SPT ecotype, while the tips of some leaves of NM ecotype showed necrosis (Additional file 12). Quinoa seedlings at six-leaf stage and wild barley seedlings at three-leaf stage were also exposed to the same heat shock conditions. The quinoa seedlings were almost killed one day after heat shock treatment and wild barley also cannot stand for this treatment (Additional file 12).

### Validation of assembled unigenes and confirmation of heat stress candidate genes by RT-PCR

Total RNA from normal leaves or heat treated leaves was isolated and 1 μg high quality RNA was reverse transcribed using the RevertAid First Strand cDNA Synthesis Kit (#K1621, ThermoFisher Scientific). The primers for the validation of assembly quality and the verification of candidate genes were designed online by BatchPrimer 3 (http://probes.pw.usda.gov/batchprimer3/) (Additional file 15). For RT-PCR, 0.2 μl of the cDNA synthesis mixture from normal leaves was used as the template, 2 μl of 10 × ExTaq buffer, 1.6 μl dNTP mixture (2.5 mM each), 0.8 μl each primer (10 μmol/l), 0.1 μl of ExTaq (5 units/μl, TaKaRa, Dalian, China) and 14.5 μl sterile distilled water were combined to a final volume of 20 μl. The PCR reaction was performed on a C1000 TOUCH thermal cycler with the following conditions: 98°C for 2 min, followed by 36 cycles of 98°C for 10 s, 55°C for 30 s, and 72°C for 1 min, and one cycle at 72°C for 10 min. The quality of the assembled unigenes was detected by loading 5 μl of the above PCR products into a 1% agarose gel along with DNA Marker 3 (TIANGEN, Beijing, China). Quantitative RT-PCR was performed on the Agilent Technologies Stratagene M × 3000P with DyNAmo Flash SYBR Green qPCR Kit (#F-415XL, ThermoFisher Scientific). The final primer concentration was 0.4 μM in 20 μl total reaction volume and 1 μl of 10 times diluted cDNA mixture was used as the templates. The reaction profile was as follows: segment 1, 95°C for 10 min; segment 2, 40 cycles of 95°C for 30 s and 60°C for 1 min; segment 3, one cycle of 95°C for 1 min, 55°C for 30 s, and 95°C for 30 s. The expression levels of candidate genes were normalized relative to that of *Actin 2* (comp237782_c0) and the levels of candidates in normal leaves were set to 1.0. Each RNA sample was assayed in triplicates and two independently biological repeats were conducted.

## Availability of supporting data

The RNA-seq raw data of this article was deposited in the NCBI Short Read Archive (SRA) under accession number SRR1559276. The sequence data supporting the phylogenetic tree of this article was deposited in the permanent and resolvable resource locator TreeBase: http://purl.org/phylo/treebase/phylows/study/TB2:S16290.

## Electronic supplementary material

Additional file 1: **Locations of red dots in Figure** 1 **.** (DOCX 49 KB)

Additional file 2: **The morphology of the sand rice adult plant.** (PPTX 352 KB)

Additional file 3: **Summary of Illumina transcriptome sequencing for Sand rice.** (DOCX 17 KB)

Additional file 4: **Overview of sand rice transcriptome sequencing and assembly.** Length distribution of Contigs (A) and transcripts (B). (C) The correlation between Unigene length and reads number assembled into the correspongding Unigenes. (D) Size distribution of Sand rice open reading frames (ORFs). (PPTX 321 KB)

Additional file 5: **Length distribution of Open Reading Frames (ORFs).** (DOCX 17 KB)

Additional file 6: **Validation of the quality of RNA-seq by RT-PCR.** Eighteen candidate genes were randomly selected for RT-PCR and 5 μl of the PCR products were loaded. *Actin 2* was used as a control. M: marker 3; White arrow showed the theory band of comp264744_c0. Primer sequences were listed in Additional file 15. (PPTX 204 KB)

Additional file 7: **GC content analysis of sand rice unigenes.** (A) Frequency of GC content of sand rice unigenes. (B) Distribution of GC content of unigenes for sand rice (As) and transcripts for *Arabidopsis* (At), Soybean (Gm), and rice (Os). (PPTX 165 KB)

Additional file 8: **Summary of unigene annotations.** Integrated function annotation and Nr species distribution, GO, COG, and KEGG annotations were shown. (XLSX 6 MB)

Additional file 9: **Characteristics of sand rice unigenes hitted deposited sequences in Nr database and newly sequenced sugar beet peptide sequences.** (A) Nr annotation results distributed by the E-value. (B) Nr annotation results based on sequence identities. (PPTX 80 KB)

Additional file 10: **Most highly represented GO terms in the sand rice trancriptome annotation.** A total of 22,270 unigenes were assigned into three main categories: Cellular component, Molecular function, and Biological process. The top 50 represented terms were represented. (PPTX 323 KB)

Additional file 11: **Summary of pairs of putative orthologues between sand rice and sugar beet.** Additional file 11a, Top BLASTx results of sand rice unigenes versus sugar beet proteins. Additional file 11b, Top tBLASTn results of sugar beet proteins versus sand rice unigenes. Additional file 11c, Results of pairs of putative orthologs between sand rice and sugar beet. Additional file 11d, GO annotations for sand rice unigenes with sugar beet orthlogs. A total of 11,581 unigenes was assigned with at least one GO term. Additional file 11e, Statistical summary of GO categories for doublet unigenes. Additional file 11f, Swissprot and Nr annotations of 782 unigenes categorized into response to salt stress term. (XLSX 9 MB)

Additional file 12: **Thermoltolerance assays.** (A) Sand rice seedlings were exposed to 50°C for 3 h in dark and then moved back to greenhouse to recovery for 7 days. The upper panel was SPT ecotype and the bottom panel was NM ecotype. (B) Quinoa and barley (C) seedlings were exposed to the same heat shock treatment. (PPTX 151 KB)

Additional file 13: **Summary of candidate genes in sand rice trancriptome.** Additional file 13a, Swissprot and Nr annotations of the 1000 most highly expressed unigenes. Additional file 13b, The 26 putative kinase among the 1000 most highly expressed unigenes. There are 19 unigenes with orthologs in *Arabidopsis*. Additional file 13c, The 24 putative transcription factors among the 1000 most highly expressed unigenes. There are 21 unigenes with orthologs in *Arabidopsis*. Additional file 13d, Statistical summary of GO categories of the 1000 most highly expressed unigenes. Additional file 13e, Swissprot and Nr annotations of 43 unigenes categorized into response to heat term. The putative HSPs are highlighted in yellow. Additional file 13f, Summary of 33 putative HSP unigenes with RBH in *Arabidopsis*. The HSP genes list and subcellular location are derived from Finka et al. [50]. The twelve unigenes include into the top 1000 accumulated unigenes are showed with red font and seven unigene assigned into response to heat term are highlighted in purple. Additional file 13g, Summary of 12 putative HSF unigenes with RBH in *Arabidopsis*. Additional file 13h, Summary of possible heat stress-related and -specific genes in sand rice transcriptome. The *Arabidopsis* genes information was derived from Ma et al. [57, 58]. Additional file 13i, Summary of possible multiple stress specific genes in sand rice transcriptome. The *Arabidopsis* genes information was derived from Sewelam et al. [62]. The unigenes included in the top 1000 expressed category were highlighted in yellow. (XLSX 296 KB)

Additional file 14: **Detection of heat stress candidate genes by qRT-PCR.** Sand rice seedlings with five leaves were subjected to heat stress (50°C) treatment and the control condition for 3 h, and then RNA was extracted from normal and heat-stressed leaves to perform qRT-PCR. The expression levels of candidates were normalized relative to that of *Actin 2* (comp237782_c0) and the levels of candidates in normal leaves were set to 1.0. Each RNA sample was assayed in triplicates and two independently biological repeats were conducted. (A) *SnRK2.4*; (B) *HsfA1b*; *HSPs* (C-E); heat stress–related gene (F), and heat stress–specific genes (C, E, and G); (H) *LTP4*. (PPTX 86 KB)

Additional file 15: **Primers for the validation of assembly quality and the verification of candidate genes.** (DOCX 20 KB)

## Abbreviations

GO: Gene ontology
RBH: Reciprocal best hits
MBF1c: Transcriptional coactivator multiprotein bridging factor 1c
HSP: Heat-shock protein
HSF: Heat-shock transcription factors.
