# Supplementary material for: Transcriptomic analysis of a psammophyte food crop, sand rice (Agriophyllum squarrosum) and identification of candidate genes essential for sand dune adaptation
Source: BMC Genomics. 2014 Oct 7;15(1):872. doi: 10.1186/1471-2164-15-872 (PMC4459065; doi:10.1186/1471-2164-15-872)
Supplement: Supplementary file 1 — Additional file 1: Locations of red dots in Figure 1 . (DOCX 49 KB) [file 12864_2014_7070_MOESM1_ESM.docx]

Additional file 1. Locations of red dots in Figure 1.

| Names of red dots | Longitude (^°^) | Latitude (^°^) |
| --- | --- | --- |
| MQXX | 103.34 | 38.89 |
| ML | 90.12 | 44.27 |
| WLJ | 104.57 | 40.65 |
| YG | 105.19 | 40.83 |
| AYQ | 101.68 | 39.25 |
| JLT | 105.68 | 39.93 |
| JCK | 105.60 | 39.52 |
| BLG | 107.03 | 40.26 |
| DL | 98.12 | 36.31 |
| M1 | 101.49 | 39.64 |
| QHH | 100.79 | 36.72 |
| BEJX | 86.61 | 47.66 |
| FK | 88.14 | 44.37 |
| TGX | 100.57 | 36.17 |
| HJZHE | 108.86 | 40.49 |
| KY | 108.97 | 39.41 |
| JT | 94.00 | 39.96 |
| DH | 94.67 | 40.11 |
| XSW | 109.96 | 40.24 |
| BYWS | 108.58 | 39.94 |
| DLSH | 108.59 | 41.27 |
| DSHT | 108.98 | 40.94 |
| SSG | 94.37 | 39.66 |
| JHXN | 82.91 | 44.48 |
| SPT | 105.00 | 37.48 |
| YLH | 105.35 | 38.75 |
| M5 | 102.54 | 39.82 |
| M2 | 102.79 | 39.73 |
| M4 | 101.43 | 40.64 |
| M6 | 102.16 | 39.75 |
| XJ | 103.69 | 37.55 |
| LJT | 105.33 | 37.95 |
| S136 | 100.56 | 39.73 |
| TGL | 105.02 | 37.57 |
| WSQ | 108.85 | 38.56 |
| YLZBJ | 91.60 | 29.25 |
| AKS | 94.21 | 39.42 |
| BB | 107.60 | 37.61 |
| JB | 108.74 | 37.62 |
| MMH | 106.83 | 38.84 |
| HJQ | 109.28 | 39.85 |
| NM | 120.65 | 42.87 |
