# Supplementary figures and images for: Transcriptomic analysis of a psammophyte food crop, sand rice (Agriophyllum squarrosum) and identification of candidate genes essential for sand dune adaptation
Source: BMC Genomics. 2014 Oct 7;15(1):872. doi: 10.1186/1471-2164-15-872 (PMC4459065; doi:10.1186/1471-2164-15-872)

## Slide 1
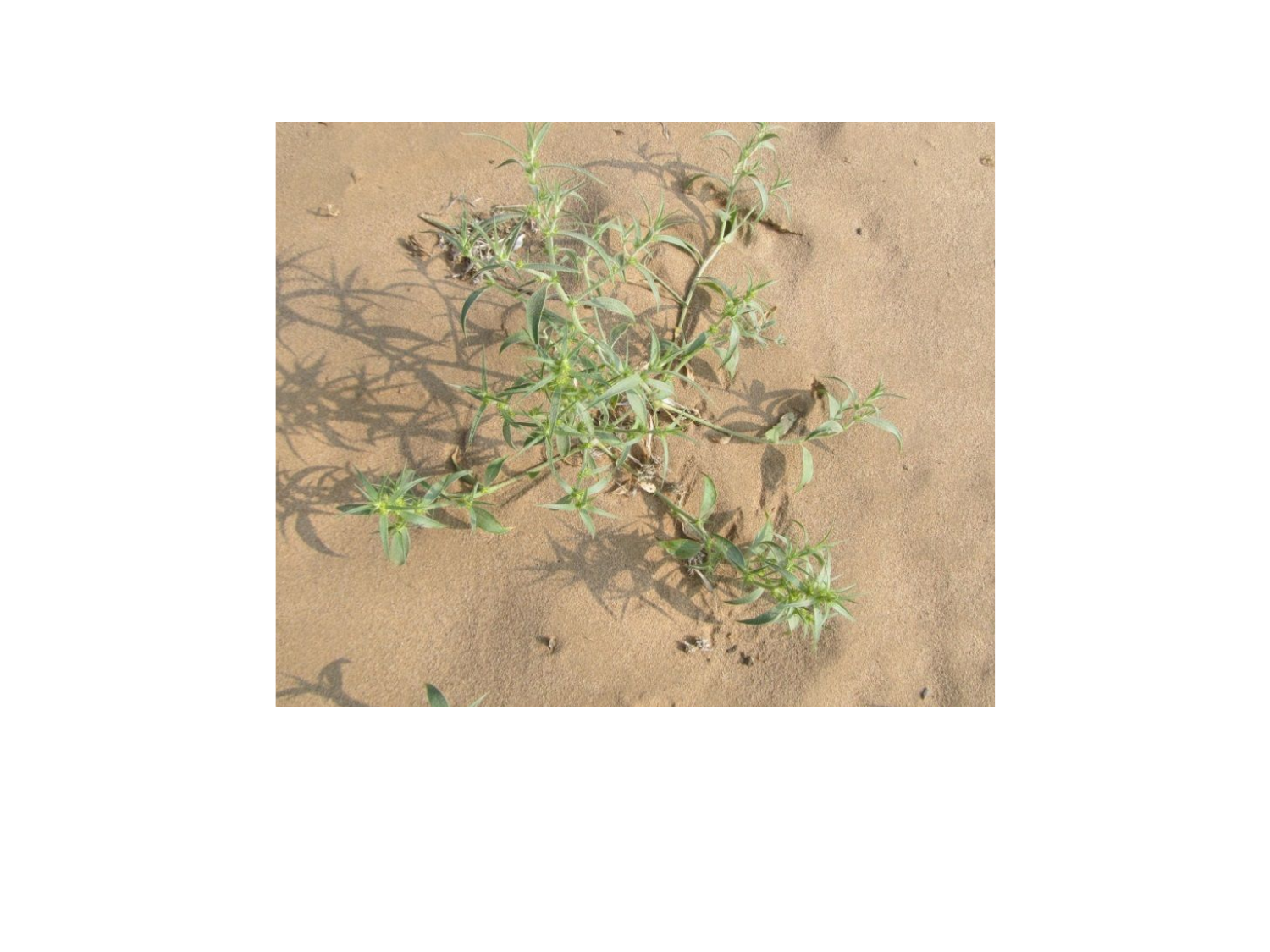

Supplement: Supplementary file 2 — Additional file 2: The morphology of the sand rice adult plant. (PPTX 352 KB) [file 12864_2014_7070_MOESM2_ESM.pptx]

## Slide 1
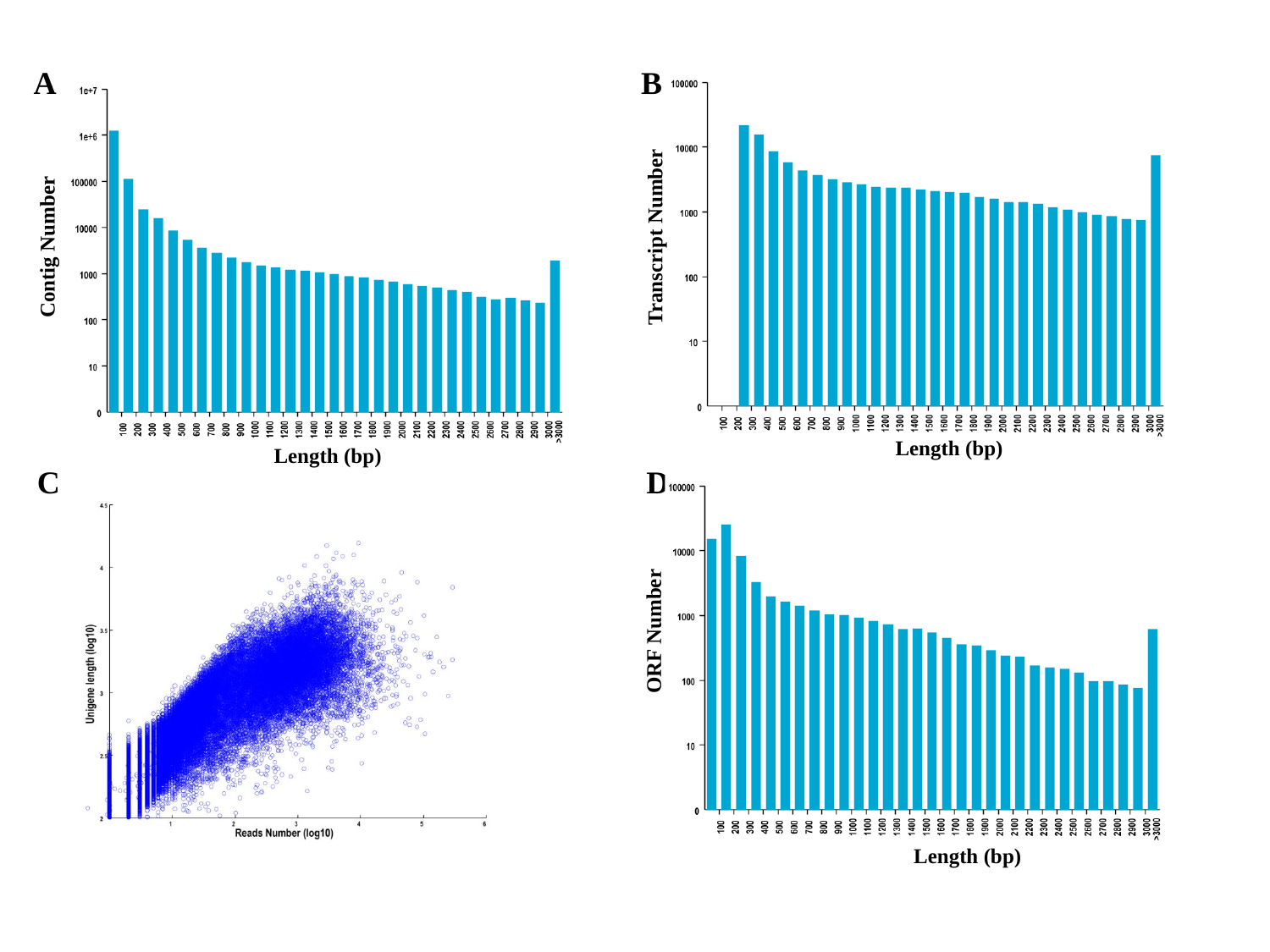

A B
Transcript Number
Contig Number
Length (bp)
Length (bp)
C D
ORF Number
Length (bp)

Supplement: Supplementary file 4 — Additional file 4: Overview of sand rice transcriptome sequencing and assembly. Length distribution of Contigs (A) and transcripts (B). (C) The correlation between Unigene length and reads number assembled into the correspongding Unigenes. (D) Size distribution of Sand rice open reading frames (ORFs). (PPTX 321 KB) [file 12864_2014_7070_MOESM4_ESM.pptx]

## Slide 1
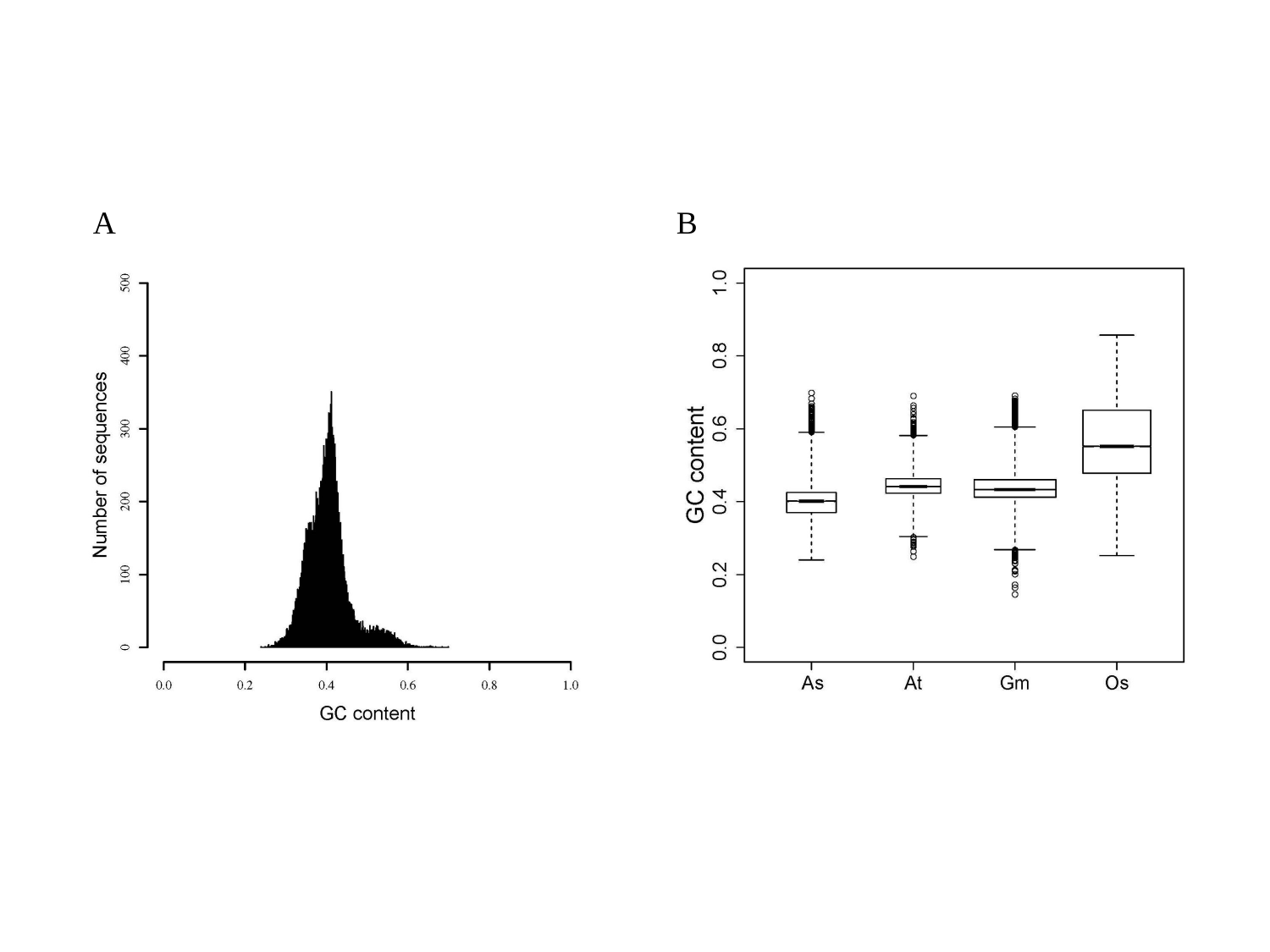

A B

Supplement: Supplementary file 7 — Additional file 7: GC content analysis of sand rice unigenes. (A) Frequency of GC content of sand rice unigenes. (B) Distribution of GC content of unigenes for sand rice (As) and transcripts for Arabidopsis (At), Soybean (Gm), and rice (Os). (PPTX 165 KB) [file 12864_2014_7070_MOESM7_ESM.pptx]

## Slide 1
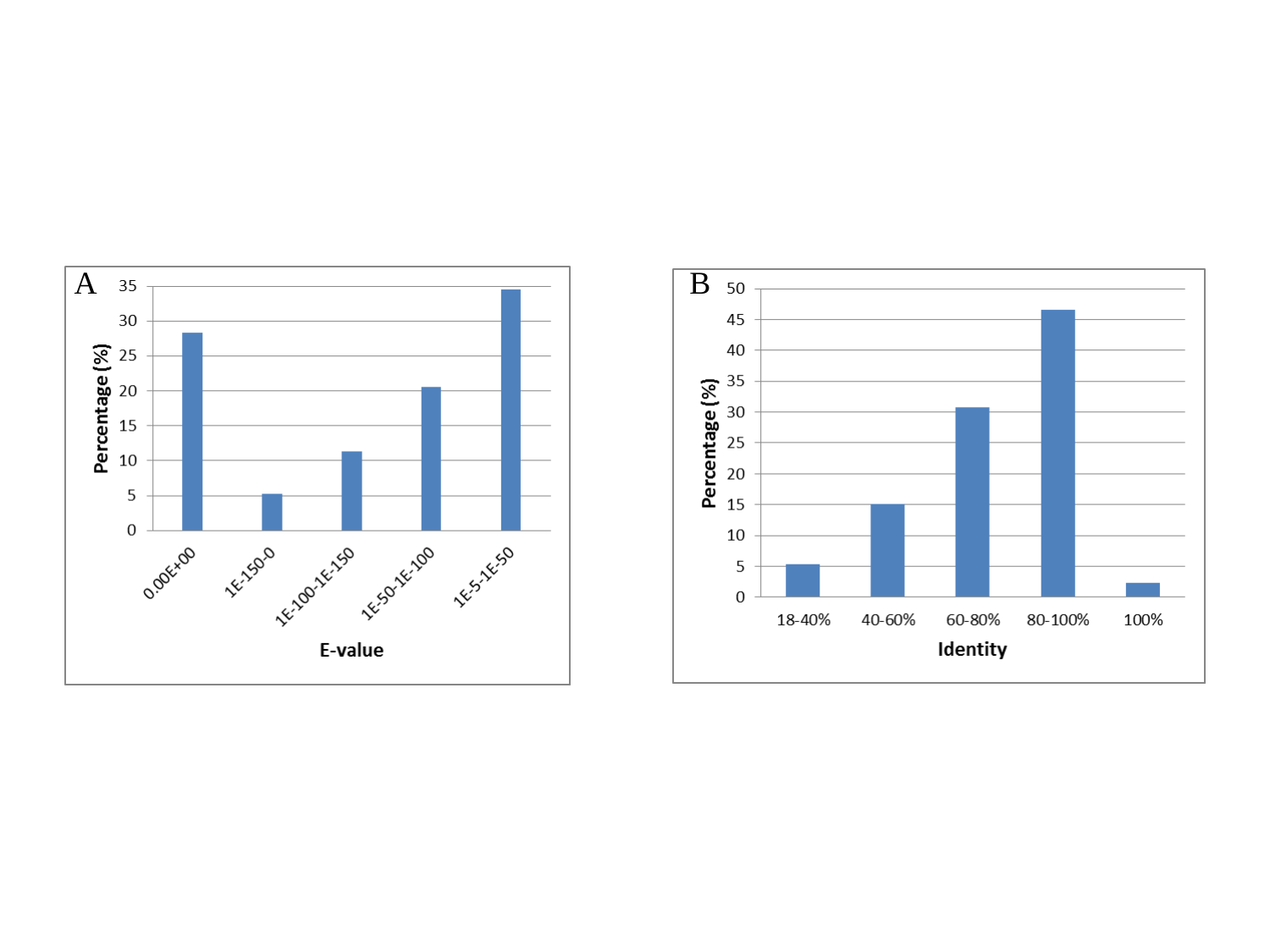

A B

Supplement: Supplementary file 9 — Additional file 9: Characteristics of sand rice unigenes hitted deposited sequences in Nr database and newly sequenced sugar beet peptide sequences. (A) Nr annotation results distributed by the E-value. (B) Nr annotation results based on sequence identities. (PPTX 80 KB) [file 12864_2014_7070_MOESM9_ESM.pptx]

## Slide 1
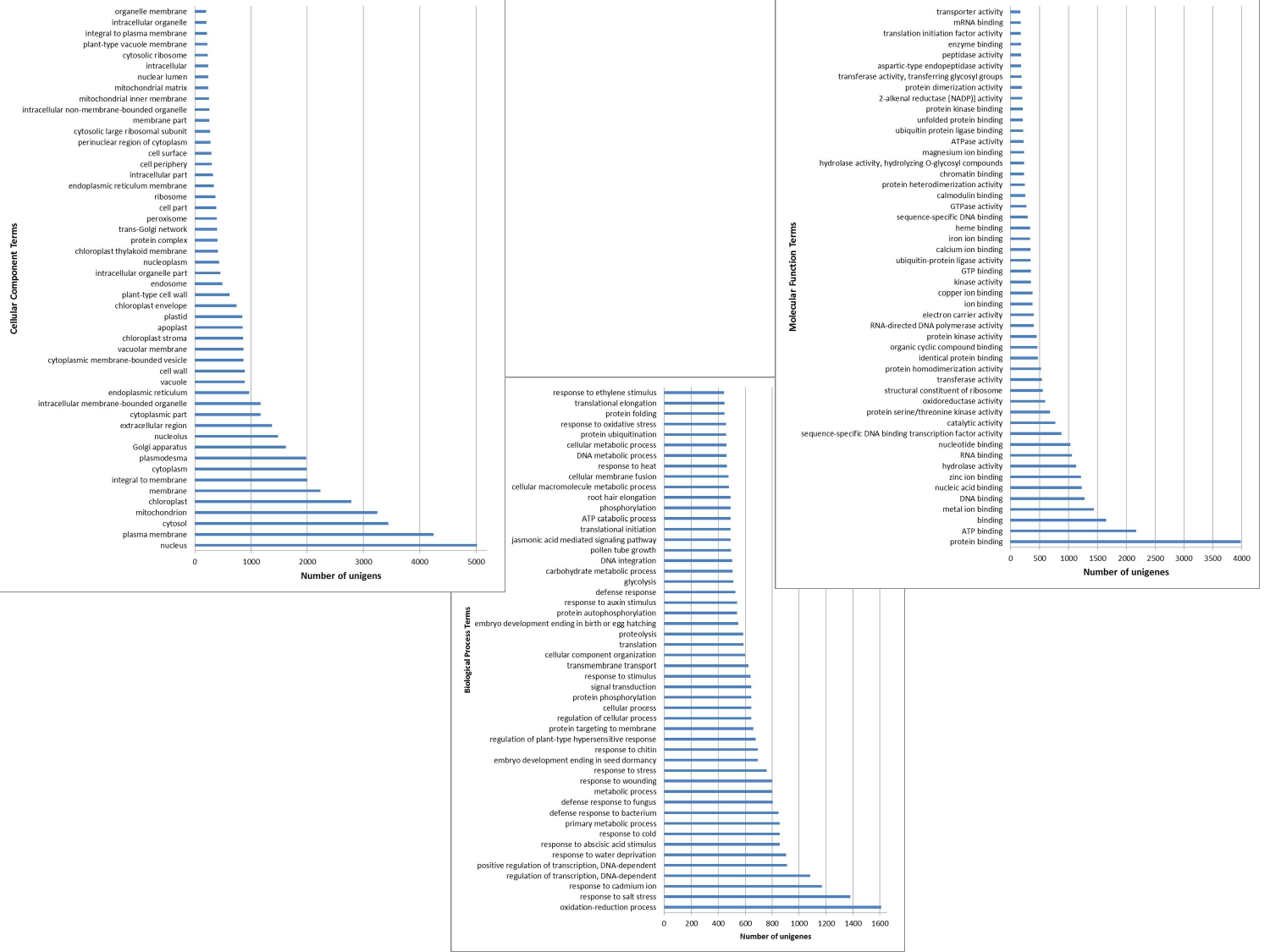

Supplement: Supplementary file 10 — Additional file 10: Most highly represented GO terms in the sand rice trancriptome annotation. A total of 22,270 unigenes were assigned into three main categories: Cellular component, Molecular function, and Biological process. The top 50 represented terms were represented. (PPTX 323 KB) [file 12864_2014_7070_MOESM10_ESM.pptx]

## Slide 1
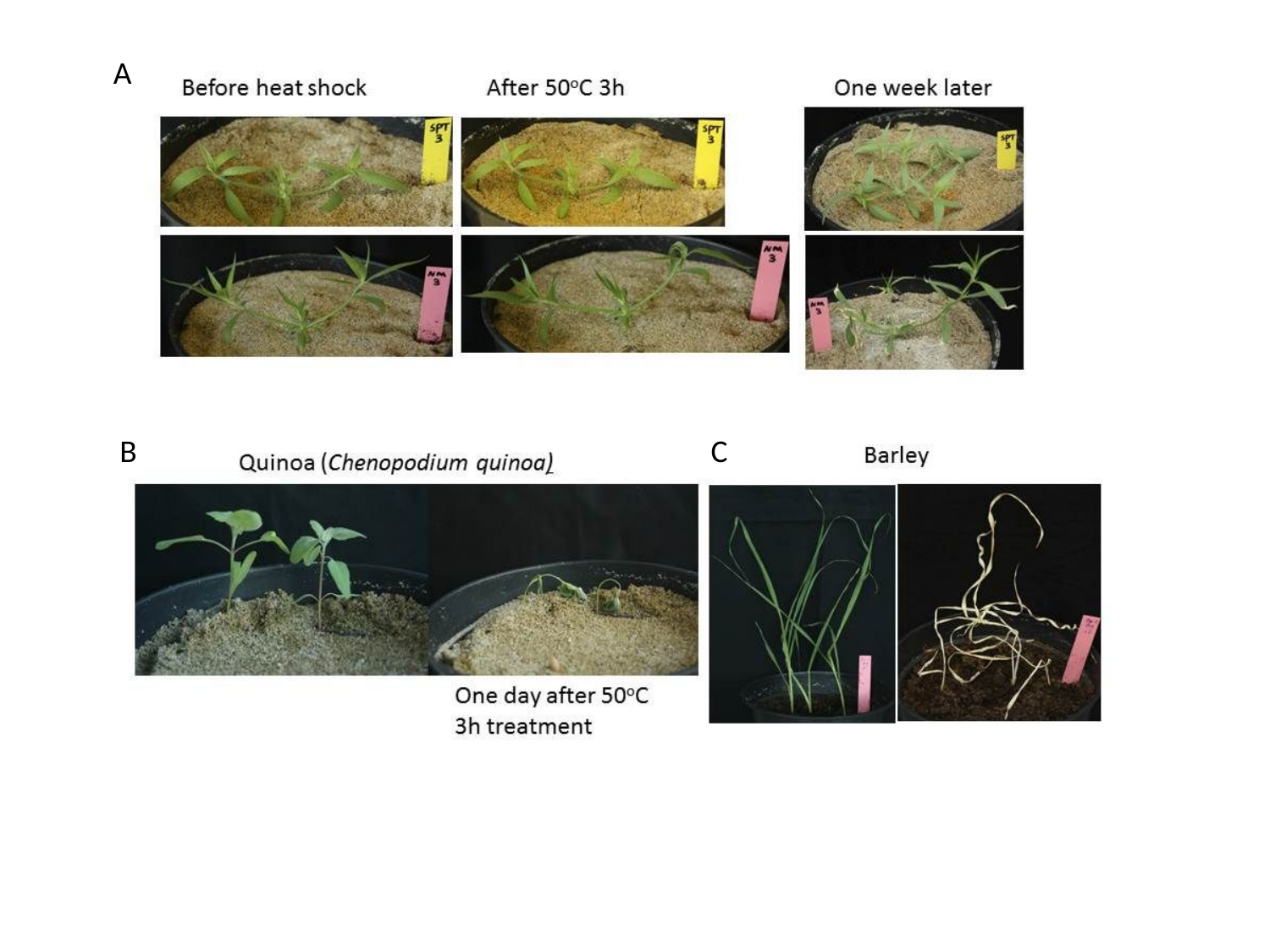

A
B C

Supplement: Supplementary file 12 — Additional file 12: Thermoltolerance assays. (A) Sand rice seedlings were exposed to 50°C for 3 h in dark and then moved back to greenhouse to recovery for 7 days. The upper panel was SPT ecotype and the bottom panel was NM ecotype. (B) Quinoa and barley (C) seedlings were exposed to the same heat shock treatment. (PPTX 151 KB) [file 12864_2014_7070_MOESM12_ESM.pptx]
