# Supplementary material for: Transcriptomic analysis of a psammophyte food crop, sand rice (Agriophyllum squarrosum) and identification of candidate genes essential for sand dune adaptation
Source: BMC Genomics. 2014 Oct 7;15(1):872. doi: 10.1186/1471-2164-15-872 (PMC4459065; doi:10.1186/1471-2164-15-872)
Supplement: Supplementary file 3 — Additional file 3: Summary of Illumina transcriptome sequencing for Sand rice. (DOCX 17 KB) [file 12864_2014_7070_MOESM3_ESM.docx]

Additional file 3. Summary of Illumina transcriptome sequencing for Sand rice.

| Samples | Total reads | Total nucleotides (bp) | GC percentage | Q30 percentage |
| --- | --- | --- | --- | --- |
| Sand rice | 30,283,868 | 6,100,519,173 | 45.32% | 86.88% |
