# Supplementary material for: Transcriptomic analysis of a psammophyte food crop, sand rice (Agriophyllum squarrosum) and identification of candidate genes essential for sand dune adaptation
Source: BMC Genomics. 2014 Oct 7;15(1):872. doi: 10.1186/1471-2164-15-872 (PMC4459065; doi:10.1186/1471-2164-15-872)
Supplement: Supplementary file 5 — Additional file 5: Length distribution of Open Reading Frames (ORFs). (DOCX 17 KB) [file 12864_2014_7070_MOESM5_ESM.docx]

Additional file 5. Length distribution of Open Reading Frames (ORFs).

| Length range (bp) | Unigene ORF |
| --- | --- |
| 0-300 | **48,480 (71.87%)** |
| 300-500 | **5,175 (7.67%)** |
| 500-1000 | **6,158 (9.13%)** |
| 1000-2000 | **5,629 (8.34%)** |
| >2000 | **2,016 (2.99%)** |
| Total number | **67,458** |
| Total length | **26,874,744** |
| N50 length | **1,029** |
| Mean length | **398.39** |
