# Supplementary material for: Transcriptomic analysis of a psammophyte food crop, sand rice (Agriophyllum squarrosum) and identification of candidate genes essential for sand dune adaptation
Source: BMC Genomics. 2014 Oct 7;15(1):872. doi: 10.1186/1471-2164-15-872 (PMC4459065; doi:10.1186/1471-2164-15-872)
Supplement: Supplementary file 6 — Additional file 6: Validation of the quality of RNA-seq by RT-PCR. Eighteen candidate genes were randomly selected for RT-PCR and 5 μl of the PCR products were loaded. Actin 2 was used as a control. M: marker 3; White arrow showed the theory band of comp264744_c0. Primer sequences were listed in Additional file 15. (PPTX 204 KB) [file 12864_2014_7070_MOESM6_ESM.pptx]

## Slide 1
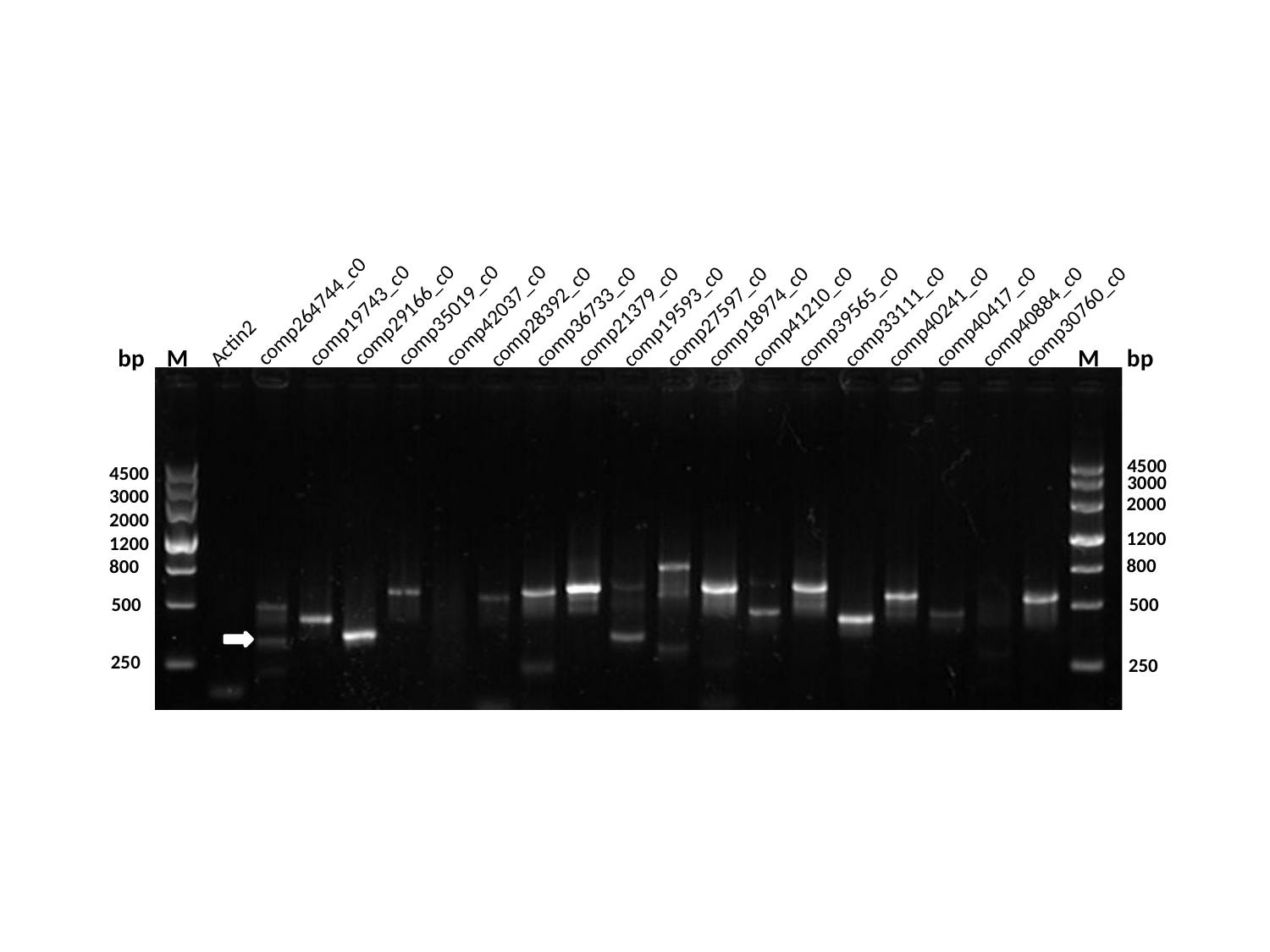

comp264744_c0
comp29166_c0
comp35019_c0
comp42037_c0
comp19743_c0
comp28392_c0
comp36733_c0
comp21379_c0
comp19593_c0
comp27597_c0
comp18974_c0
comp41210_c0
comp39565_c0
comp33111_c0
comp40241_c0
comp40417_c0
comp40884_c0
comp30760_c0
Actin2
bp M
M bp
4500
4500
3000
2000
1200
800
3000
2000
1200
800
500
500
250
250
