# Supplementary material for: Transcriptomic analysis of a psammophyte food crop, sand rice (Agriophyllum squarrosum) and identification of candidate genes essential for sand dune adaptation
Source: BMC Genomics. 2014 Oct 7;15(1):872. doi: 10.1186/1471-2164-15-872 (PMC4459065; doi:10.1186/1471-2164-15-872)
Supplement: Supplementary file 14 — Additional file 14: Detection of heat stress candidate genes by qRT-PCR. Sand rice seedlings with five leaves were subjected to heat stress (50°C) treatment and the control condition for 3 h, and then RNA was extracted from normal and heat-stressed leaves to perform qRT-PCR. The expression levels of candidates were normalized relative to that of Actin 2 (comp237782_c0) and the levels of candidates in normal leaves were set to 1.0. Each RNA sample was assayed in triplicates and two independently biological repeats were conducted. (A) SnRK2.4; (B) HsfA1b; HSPs (C-E); heat stress–related gene (F), and heat stress–specific genes (C, E, and G); (H) LTP4. (PPTX 86 KB) [file 12864_2014_7070_MOESM14_ESM.pptx]

## Slide 1
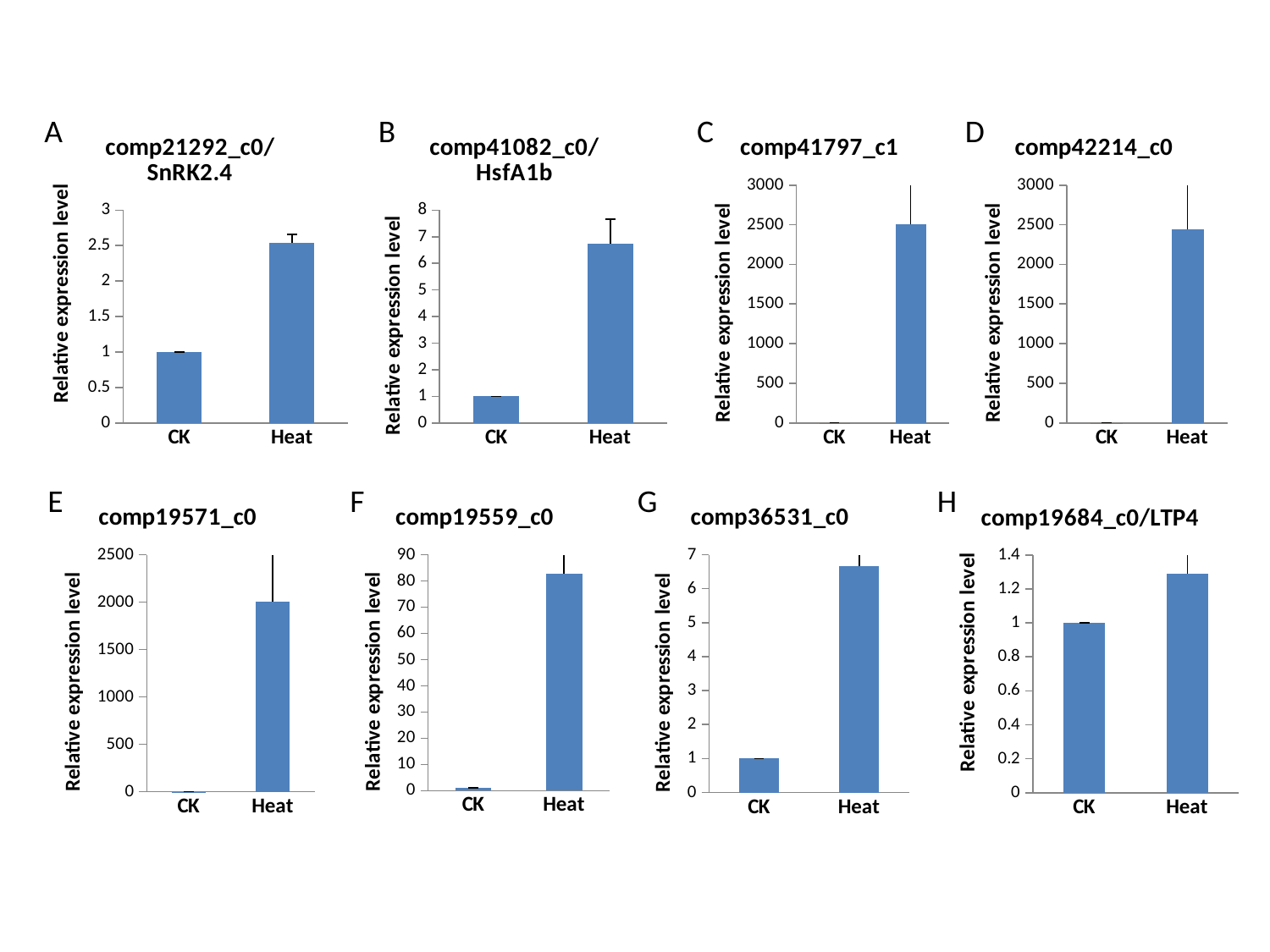

A B C D
### Chart: comp21292_c0/SnRK2.4
| Category | |
|---|---|
| CK | 1.0 |
| Heat | 2.5358618463874865 |
### Chart: comp41797_c1
| Category | |
|---|---|
| CK | 1.0 |
| Heat | 2501.863412384024 |
### Chart: comp41082_c0/HsfA1b
| Category | |
|---|---|
| CK | 1.0 |
| Heat | 6.736243969316643 |
### Chart: comp42214_c0
| Category | |
|---|---|
| CK | 1.0 |
| Heat | 2441.583525387013 | E F G H
### Chart: comp19571_c0
| Category | |
|---|---|
| CK | 1.0 |
| Heat | 2007.6629085175898 |
### Chart: comp19559_c0
| Category | |
|---|---|
| CK | 1.0 |
| Heat | 82.80580051039445 |
### Chart: comp36531_c0
| Category | |
|---|---|
| CK | 1.0 |
| Heat | 6.65852163004398 |
### Chart: comp19684_c0/LTP4
| Category | |
|---|---|
| CK | 1.0 |
| Heat | 1.288603085024523 |
