# Supplementary material for: Transcriptomic analysis of a psammophyte food crop, sand rice (Agriophyllum squarrosum) and identification of candidate genes essential for sand dune adaptation
Source: BMC Genomics. 2014 Oct 7;15(1):872. doi: 10.1186/1471-2164-15-872 (PMC4459065; doi:10.1186/1471-2164-15-872)
Supplement: Supplementary file 15 — Additional file 15: Primers for the validation of assembly quality and the verification of candidate genes. (DOCX 20 KB) [file 12864_2014_7070_MOESM15_ESM.docx]

Additional file 15a. Primers for the validation of the quality of RNA-seq.

| Gene ID | Unigene length | Primer name | Primer sequences |
| --- | --- | --- | --- |
| comp237782_c0  (actin2) | 250 | comp237782_c0_8 | GGTATGTGCAAAGCCGGATT |
|  |  | comp237782_c0_121 | TGAGTCTTTCTGGCCCATGC |
| comp264744_c0 | 582 | comp264744_c0_269 | GCTGTGAGATTCTTGGGCAATA |
|  |  | comp264744_c0_572 | GCTTGGGTATTCTGTGCAGT |
| comp19743_c0 | 700 | comp19743_c0_230 | TGAAGCACTTGAGGGGATGT |
|  |  | comp19743_c0_624 | ATCCAAAGCTACTCCTCCGC |
| comp29166_c0 | 939 | comp29166_c0_237 | GAAATCTCCAATCAGCCCGG |
|  |  | comp29166_c0_553 | TGCTTAACCTCCTCCTCAGC |
| comp35019_c0 | 1195 | comp35019_c0_478 | TTTCACGCTGCTGGTTCAAA |
|  |  | comp35019_c0_1055 | CTCATCTTCCTAACCCGCCA |
| comp42037_c0 | 1338 | comp42037_c0_410 | CGGTGATGGGAGATTAGGGG |
|  |  | comp42037_c0_997 | TCAAGCAAGCAATCACCGTG |
| comp28392_c0 | 1672 | comp28392_c0_16 | CTGTTCTTCCCCTCCCTCAG |
|  |  | comp28392_c0_547 | CTCCCATGGCTCGATCATCT |
| comp36733_c0 | 1742 | comp36733_c0_1111 | ATCCGGCCTGATATGGTGAG |
|  |  | comp36733_c0_1704 | TAAATCCACGCCCACTCCAT |
| comp21379_c0 | 2054 | comp21379_c0_768 | AAATGTTGCACTGGTAGGCC |
|  |  | comp21379_c0_1374 | AGGATCACGACCCACAATGT |
| comp19593_c0 | 2138 | comp19593_c0_326 | CCAGACAAGCTACCACCTCA |
|  |  | comp19593_c0_962 | AAGACCAATTGATGCAGCCC |
| comp27597_c0 | 2262 | comp27597_c0_1104 | GCTGCTGAGATTGATGAGGC |
|  |  | comp27597_c0_1762 | AGTTGAGCACACTAGGGTCC |
| comp18974_c0 | 2437 | comp18974_c0_1380 | GGCCAGGGGAAGTTTTGAAG |
|  |  | comp18974_c0_2044 | ACACGCAGAAACACGAAACA |
| comp41210_c0 | 2599 | comp41210_c0_2042 | TCCACGTCAAAGTCAGAGCT |
|  |  | comp41210_c0_2485 | ACCCCACCATCTTCACCAAA |
| comp39565_c0 | 3387 | comp39565_c0_1300 | GAGTCTCAACGTGGTGATGC |
|  |  | comp39565_c0_1962 | CTGACGTATACCCGCAGCTA |
| comp33111_c0 | 4413 | comp33111_c0_32 | AAGGCCCATGAGAACCAGAA |
|  |  | comp33111_c0_446 | CAGCATAGCCAATCACCACC |
| comp40241_c0 | 5278 | comp40241_c0_4341 | CATCTTGAGCCGAGCACATC |
|  |  | comp40241_c0_4920 | CATTCTGCAGGCACATCTCC |
| comp40417_c0 | 5474 | comp40417_c0_987 | GTGCGTTCTTGTCGTTCACT |
|  |  | comp40417_c0_1449 | CCGAGATCCAACCTCCCAAT |
| comp40884_c0 | 6556 | comp40884_c0_663 | CCTCCACATTGTCACTCCCT |
|  |  | comp40884_c0_1216 | CGCAACCTCAGTGAACAACA |
| comp30760_c0 | 7709 | comp30760_c0_4003 | CAACCTGGCGAACTGAAACA |
|  |  | comp30760_c0_4596 | TCAACATCAGTCGGTTCGGA |

Additional file 15b. Primers for the confirmation of heat stress candidate genes by qRT-PCR.

| comp21292_c0 | comp21292_c0_377 | GGTTTTGACACCAACCCATC |
| --- | --- | --- |
|  | comp21292_c0_528 | TGGAGTGGCAGAAGCTAACA |
| comp41082_c0 | comp41082_c0_233 | ATGGGAGAATCGTCGTTGAC |
|  | comp41082_c0_378 | ACACTCCAGGAAACCACAGC |
| comp41797_c1 | comp41797_c1_554 | TGGATGAGGTTAAGGCCAAC |
|  | comp41797_c1_704 | AATCCACCCCATTTGAACAC |
| comp42214_c0 | comp42214_c0_201 | GGTAGACCGACCCCCTAGTC |
|  | comp42214_c0_348 | CTTCCAGTCCACCCTAGCAG |
| comp19571_c0 | comp19571_c0_97 | TTTCTTGCCCTTCACCTCAC |
|  | comp19571_c0_245 | TGAGCCTAAGAAGCCCAAGA |
| comp19559_c0 | comp19559_c0_3021 | ATCACGAGGCTTCTCCTCAA |
|  | comp19559_c0_3169 | TGCAATTTCCACCAGAACAA |
| comp36531_c0 | comp36531_c0_485 | TCTTGAGGAGGCTGATCGTT |
|  | comp36531_c0_634 | GCACCGCTGATATGGAAACT |
| comp19684_c0 | comp19684_c0_241 | CCACTAGCCTTGCTCCATGT |
|  | comp19684_c0_388 | GCTGATTTGAGGCATTGACA |
